# COL10A1^+^ fibroblasts define rupture-prone abdominal aortic aneurysms and enable molecular risk stratification

**DOI:** 10.1186/s12967-026-08689-8

**Published:** 2026-07-23

**Authors:** Huawei Zhuo, Chengxin Ou, Meng Duan, Jiwu Yang, Dan Liu

**Affiliations:** 1 Department of Vascular Surgery, Xuzhou Central Hospital, Xuzhou, China; 2 Xuzhou Clinical School of Xuzhou Medical University, Xuzhou, China; 3 Cancer Institute, Cellular Therapeutics School of Medicine, Xuzhou Medical University, 209 Tongshan Road, Xuzhou, Jiangsu 221004 China; 4 Center of Clinical Oncology, The Affiliated Hospital of Xuzhou Medical University, 99 West Huaihai Road, Xuzhou, Jiangsu 221002 China; 5 Jiangsu Center for the Collaboration and Innovation of Cancer Biotherapy, Xuzhou Medical University, 209 Tongshan Road, Xuzhou, Jiangsu 221004 China

**Keywords:** Abdominal aortic aneurysm, Fibroblast heterogeneity, COL10A1, Single-cell RNA sequencing, Vascular microenvironment, Risk stratification

## Abstract

**Background:**

Abdominal aortic aneurysm (AAA) rupture remains the leading cause of AAA-related mortality, while current clinical management relies primarily on aneurysm diameter, which inadequately predicts rupture risk. Increasing evidence suggests that stromal cell heterogeneity critically shapes vascular remodeling; however, the specific fibroblast states associated with AAA rupture predisposition remain poorly defined.

**Methods:**

We integrated cross-species single-cell RNA-sequencing datasets spanning the full spectrum of AAA progression to systematically characterize fibroblast heterogeneity and state transitions. Pseudotime trajectory analysis, transcriptional regulatory network inference, and ligand–receptor interaction modeling were applied to delineate fibroblast-associated microenvironmental programs. Based on these findings, a fibroblast-derived molecular signature was constructed and validated in an independent human AAA cohort.

**Results:**

Single-cell analysis revealed a progressive transition from homeostatic IGFBP6^+^ fibroblasts to a disease-associated COL10A1^+^ fibroblast state during AAA progression. COL10A1^+^ fibroblasts were selectively enriched in rupture-prone lesions and exhibited transcriptional programs linked to extracellular matrix remodeling and osteogenic-like activation. Intercellular communication analysis indicated that COL10A1^+^ fibroblasts were predicted to engage macrophage populations through coordinated ligand–receptor signaling pathways, including MDK, CSF1, and CXCL12, collectively delineating a rupture-associated vascular microenvironment. Translationally, leveraging this fibroblast state enabled the development of a minimal two-gene risk score (COL10A1–IGFBP6), which stratified AAA patients by molecular rupture risk independently of aneurysm diameter in the validation cohort.

**Conclusions:**

This study identifies a COL10A1^+^ fibroblast–associated transcriptional state that defines a rupture-prone vascular microenvironment in abdominal aortic aneurysm. The derived COL10A1–IGFBP6 signature establishes a biologically informed framework for molecular risk stratification, complementing anatomical size–based assessment and providing a foundation for future development of mechanism-guided strategies to refine AAA risk evaluation.

**Supplementary Information:**

The online version contains supplementary material available at https://doi.org/10.1186/s12967-026-08689-8.

## Key points

Single-cell analysis identifies a disease-associated COL10A1^+^ fibroblast state enriched in abdominal aortic aneurysms (AAA) with features associated with rupture.

COL10A1^+^ fibroblasts are linked to extracellular matrix remodeling and macrophage crosstalk, defining a rupture associated microenvironment.

The COL10A1–IGFBP6 molecular signature stratifies AAA patients by molecular rupture risk, independent of aneurysm diameter.

This study establishes a biologically informed framework that complements current anatomical size–based AAA risk assessment.

**Supplementary Information:**

The online version contains supplementary material available at https://doi.org/10.1186/s12967-026-08689-8.

## Introduction

Abdominal aortic aneurysm (AAA) is a major cause of cardiovascular morbidity and mortality, particularly in the aging population. It is characterized by the progressive and irreversible dilation of the infrarenal aorta, which can culminate in the catastrophic event of rupture, associated with in-hospital mortality rates of 80–90% [1, 2]. The clinical challenge lies in its asymptomatic nature prior to rupture, making the preemptive identification of high-risk patients paramount.

Elective surgical intervention for AAA is predominantly guided by the maximum aortic diameter, with established size thresholds (e.g., 55 mm for men) stemming from landmark population-based studies. However, this size-based criterion is fraught with well-recognized limitations and fails to account for individual variation in rupture risk [3]. A significant proportion of small aneurysms rupture before reaching the intervention threshold, while many large aneurysms remain stable for years [4]. This “diameter paradox” underscores that aneurysm size is an imperfect surrogate for the complex biological processes determining wall integrity and rupture susceptibility and highlights the urgent need to identify molecular and cellular drivers of disease progression [5].

The pathobiology of AAA involves a complex interplay of chronic inflammation, extracellular matrix (ECM) degradation, vascular smooth muscle cell (VSMC) depletion, and neovascularization [6]. Historically, research has focused on infiltrating immune cells, particularly macrophages and lymphocytes, as the principal mediators of this destructive microenvironment [7]. Vascular SMCs, the key cellular component of the media, have also been extensively studied for their phenotypic modulation and apoptosis in AAA [8]. In contrast, fibroblasts—the most abundant stromal cells in the vascular wall—have been largely overlooked and traditionally viewed as passive, matrix-producing support cells.

This view is being radically revised in other disease contexts, such as cancer and fibrosis, where fibroblasts are now recognized as dynamic, functionally heterogeneous regulators of immunity, fibrosis, and tissue remodeling [9, 10]. Of particular interest is Collagen type X alpha-1 (COL10A1), a short-chain fibrillar collagen originally identified in hypertrophic chondrocytes and classically associated with cartilage maturation and skeletal development [11]. Recent multi-omics studies have shown aberrant upregulation of COL10A1 in the tumor stroma of breast, pancreatic, and gastric cancers, where it correlates with lymph node metastasis, hematogenous spread, and poor prognosis [12, 13]. Functionally, COL10A1 contributes to ECM remodeling and interacts with proteins such as prolyl 4-hydroxylase subunit beta (P4HB) and integrin subunit beta 1 (ITGB1) to facilitate cell proliferation, migration, and tissue remodeling [11, 14]. However, the precise origin, cellular sources, and mechanistic role of COL10A1 within vascular disease contexts, such as AAA, remain largely unknown.

In the aorta, adventitial fibroblasts are uniquely positioned as biosensors and signaling hubs. They are among the first responders to hemodynamic stress and inflammatory signals from the lumen and vasa vasorum, orchestrating downstream response [15, 16]. We therefore hypothesize that within the AAA microenvironment, specialized, pathogenic fibroblast subpopulations exist that are not merely reactive but are active drivers of disease progression toward rupture.

The advent of single-cell RNA sequencing (scRNA-seq) provides an unprecedented opportunity to dissect this cellular heterogeneity and identify such culpable subsets [17]. Recent scRNA-seq studies in AAA have begun to catalog the diversity of immune cells and VSMCs [18–20]; however, a comprehensive, functional annotation of fibroblast states across the disease continuum—from early dilation to imminent rupture—remains elusive.

In this study, we propose and validate a “fibroblast-centric” paradigm for AAA pathogenesis. By integrating multi-species, multi-stage scRNA-seq datasets, we constructed a high-resolution atlas of aortic fibroblasts and aimed to answer three central questions: (i) Does functional heterogeneity exist among fibroblasts in AAA? (ii) Is there a specific fibroblast subpopulation associated with increased susceptibility to rupture? (iii) Can the molecular signature of this subpopulation be leveraged to build a molecular framework that complements and refines diameter-based risk assessment? Our work identifies a COL10A1^+^ fibroblast–associated state as a key component of a rupture-enriched vascular microenvironment and translates this insight into a biologically informed molecular risk-stratification framework with translational potential, providing a foundation for future efforts to incorporate biological features into AAA risk assessment alongside anatomical size.

## Methods and materials

### Human tissue samples and bulk RNA-Sequencing analysis

Publicly available bulk RNA-sequencing datasets of human abdominal aortic tissue were obtained from the Gene Expression Omnibus (GEO; GSE183464 [21] and GSE202267 [22]). The integrated data included samples from 11 AAA patients and 11 normal aortic tissue controls. Corresponding clinical metadata were extracted from the original publications.

For GSE202267, raw counts were converted to FPKM using the edgeR package (v4.0.16) [23]. Gene lengths were defined as the sum of merged exon lengths based on the TxDb.Hsapiens.UCSC.hg38.knownGene annotation [24]. TMM (Trimmed Mean of *M*-values) normalization was applied [25], and FPKM values were calculated using the rpkm function with adjusted library sizes. For GSE183464, FPKM values were extracted directly from the repository. Batch effects were corrected using ComBat (sva package, v3.50.0) [26] with dataset origin as batch and disease status as biological covariate. Differential expression analysis was conducted using limma (v3.54.0) [27]. Differentially expressed genes (DEGs) were identified using stringent thresholds of an adjusted *p*-value (padj) <0.05 and an absolute log₂ fold change > 1. Principal component analysis (PCA) was applied to the variance-stabilized data to assess overall transcriptomic variance and detect batch effects. Functional enrichment analysis of DEGs for Gene Ontology (GO) biological processes and Kyoto Encyclopedia of Genes and Genomes (KEGG) pathways was conducted using clusterProfiler (v4.12.0) [28], with terms considered significant at padj < 0.05.

To estimate the relative abundance of major cell types (e.g., fibroblasts, immune cells, smooth muscle cells) from the bulk transcriptomes, we applied the MCP-counter deconvolution algorithm [29] with established cell-type-specific gene signatures. Fibroblast-associated gene expression was also evaluated using a set of canonical fibroblast markers (*COL1A1*, *COL1A2*, *COL3A1*, *DCN*, *LUM*, *PDGFRA*, and *FAP*).

### Single-cell RNA-Sequencing data acquisition, quality control, and processing

Human scRNA-seq datasets were obtained from the Gene Expression Omnibus (GEO; GSE166676 [18] and GSE237230 [20]), comprising 8 AAA samples and 2 control samples from aortobifemoral bypass surgery. Additionally, a longitudinal mouse model dataset (GSE239620) [30] covering the full spectrum of AAA disease progression—spanning Normal, Angiotensin II-induced Aortic Dilation (AAD), established AAA, and three stages of Intramural Hematoma (IMH-I, IMH-II, IMH-III)—was acquired. Clinical and experimental metadata for all samples were compiled (Table S1).

All scRNA-seq datasets were processed separately for each species using Seurat (v5.1.0) in R [31]. Initial quality control was performed per sample. Cells were filtered based on: (1) number of detected genes (200–6,000), (2) total unique molecular identifier (UMI) counts < 40,000, and (3) mitochondrial gene percentage < 20% to exclude low-quality cells and potential doublets.

Filtered datasets were normalized using the “LogNormalize” method (scale factor = 10,000). Mouse and human samples were separately merged into Seurat objects without additional batch integration, as cells from different datasets showed broad overlap across major cell populations and no obvious dataset-driven segregation. Following normalization, the top 2,000 highly variable genes (HVGs) were selected for principal component analysis (PCA). The first 30 principal components (PCs), capturing the majority of biological variance as determined by an elbow plot, were used for downstream nonlinear dimensionality reduction and clustering.

### Cell clustering and annotation

Uniform Manifold Approximation and Projection (UMAP) was utilized for non-linear dimensionality reduction based on the selected PCs. Cell clustering was performed using the *FindNeighbors* and *FindClusters* functions in Seurat. To determine the optimal clustering resolution, we performed cluster stability analysis using Clustree (v0.5.1) [32] across a range of resolutions (0.1–1.0). Based on this analysis, a resolution of 0.1 was applied to the human dataset and 0.5 to the mouse dataset. These major clusters were manually annotated based on the expression of well-established canonical marker genes: *PECAM1* and *VWF* for endothelial cells (ECs); *ACTA2*, *MYH11*, and *TAGLN* for vascular smooth muscle cells (VSMCs); *CD68*, *CD14*, *FCGR3A* (CD16), *LYZ* for macrophages/monocytes; *CD3D*, *CD3E* for T cells; and *COL1A1*, *COL1A2*, *PDGFRA*, *DCN* for fibroblasts. For mouse samples, annotation followed the same principles using homologous marker genes as defined in prior studies.

### Fibroblast identification and functional profiling

In the mouse scRNA-seq dataset, four fibroblast subclusters (Fibro_1 to Fibro_4) were identified directly from global clustering, without additional reclustering. Marker genes that defined each fibroblast subcluster were identified using the *FindAllMarkers* function with the Wilcoxon rank-sum test, requiring min.pct = 0.25 and log₂ fold change > 0.25. The relative proportion of each fibroblast subcluster was calculated and compared across the six disease stages (Normal, AAD, AAA, IMH I, IMH II, IMH III).

To characterize the biological functions of each subcluster, marker genes were identified using the Wilcoxon rank-sum test with thresholds of adjusted *p*-value < 0.01 and log₂ fold change > 1. The top 150 most significant marker genes (ranked by adjusted *p*-value) were selected for Gene Ontology (GO) enrichment analysis of Biological Processes (BP) using the clusterProfiler (v4.10.1) package [28]. Enrichment results with an adjusted *p*-value < 0.05 were considered significant. Additionally, to assess pathway activity at a systems level, Gene Set Variation Analysis (GSVA) was conducted using the GSVA R package (v1.50.5) [33]. The analysis utilized the Hallmark gene sets (*n* = 50) from the Molecular Signatures Database (MSigDB, v7.5.1) [34]. The resulting GSVA enrichment scores, representing the relative activity of each pathway per sample, were visualized in a heatmap using the ComplexHeatmap package (v2.18.0) [35], focusing on the top 30 pathways displaying the greatest variance across all disease stages.

### Pseudotemporal trajectory inference of fibroblast states

To infer potential differentiation or activation trajectories and the dynamic transitions among fibroblast subclusters, pseudotemporal ordering analysis was performed using Monocle3 (v1.3.0) [36]. The fibroblast subset, along with its associated UMAP coordinates and cluster labels from Seurat, was imported into Monocle3. A trajectory graph was learned using the learn_graph function. The Fibro_2 subcluster, characterized by high expression of *IGFBP6* and other homeostatic genes, was manually selected as the trajectory root (beginning state) based on its transcriptional profile and GO analysis. Cells were then ordered along the inferred trajectory branches, and a continuous pseudotime value was assigned to each cell, quantifying its inferred progression along the developmental or activation path.

### Inference of cell-type-specific transcriptional regulatory networks

To uncover the master transcriptional regulators driving fibroblast heterogeneity, single-cell regulon activity was inferred using pySCENIC (v0.12.1) [37] in a Python (v3.9) environment. The analysis followed the standard three-step pipeline: GRN Inference: GRNBoost2 was used to construct co-expression networks and identify potential transcription factor (TF)-target gene regulatory interactions from the fibroblast expression matrix; Motif Enrichment & Regulon Pruning: RcisTarget was applied to perform motif enrichment analysis (using cisTarget databases for mouse [mm10__refseq-r80__500bp_up_and_100bp_down_tss.mc9nr.feather]) to prune the co-expression network, retaining only direct, high-confidence TF-target relationships (regulons); Regulon Activity Scoring: AUCell was used to quantify the activity of each identified regulon in every individual cell, resulting in an AUC (Area Under the Curve) score matrix.

Duplicate regulons and those with low activity were filtered out. The final binarized regulon activity matrix (AUC score > 0.1 for active) was imported into R. To identify TFs specifically active in each subcluster, Regulon Specificity Scores (RSS) were calculated for each regulon across all subclusters.

### Analysis of intercellular communication networks

Cell–cell communication was analyzed using CellChat (v1.6.0) [38] following its standard pipeline. The analysis was performed separately for human and mouse scRNA-seq datasets to address distinct biological questions.

In the human dataset, CellChat was applied to all annotated cell types to quantify outgoing signaling strength for each cell type and identify the predominant sources of specific signaling pathways, such as CXCL.

In the mouse dataset, to investigate longitudinal changes in intercellular communication during disease progression, the mouse dataset was analyzed across six stages (Normal, AAD, AAA, IMH I, IMH II, IMH III). Neuronal and low‑abundance populations were excluded prior to analysis. Major annotated cell types included fibroblast subclusters (Fibro_1 – Fibro_4), VSMCs (expressing *Acta2, Myh11*), endothelial cells (expressing *Pecam1, Cdh5*), immune cells, and endothelial‑to‑mesenchymal transition (EndoMT) cells co‑expressing endothelial and mesenchymal/chondrogenic (*Col1a1*, *Fn1*, *Sox9*) markers. Communication networks were quantified to characterize intercellular interactions, with particular focus on fibroblast–macrophage crosstalk and stage‑specific ligand–receptor signaling changes.

### Cross-species projection of Fibro_3 and ProtMac signatures onto human bulk RNA-seq

High-specificity marker genes for the Fibro_3 and ProtMac subpopulations were identified from mouse scRNA-seq data using stringent criteria (pct.1 > 0.5, pct.2 < 0.1, avg_log_2_FC > 1.0). All marker genes meeting these criteria were retained for downstream analyses and mapped to their human orthologs using Ensembl gene annotations. Two independent human AAA bulk RNA-seq datasets (GSE202267 and GSE183464) were integrated and batch-corrected using the ComBat algorithm. For each human sample, a weighted signature score was calculated as the sum of orthologous gene expression values weighted by their corresponding mouse log_2_ fold-change values.

### Development and validation of a fibroblast-based molecular risk score

A fibroblast‑centric molecular risk score was defined as the standardized expression of COL10A1 minus the standardized expression of IGFBP6: Risk Score = Z*(COL10A1*) – Z*(IGFBP6*). The score was validated in an independent human cohort (GSE57691 [39]; *n* = 59 individuals, including 10 normal aortas, 20 small AAAs, and 29 large AAAs) using receiver‑operating‑characteristic (ROC) curve analysis. The area under the curve (AUC) was calculated with 95% confidence intervals via 2,000 bootstrap replicates. The predictive performance of the risk score was statistically compared with that of the maximum aortic diameter using DeLong’s test for two correlated ROC curves [40].

### Statistical analysis

All statistical analyses were performed using R (v4.3.1). Data distribution was assessed using the Shapiro–Wilk test. Data are presented as mean ± SD or median (IQR) as appropriate. For two-group comparisons, Welch’s two-tailed t-test or the Mann–Whitney U test was used as appropriate. For multiple-group comparisons, one-way ANOVA followed by Tukey’s post hoc test or the Kruskal–Wallis test followed by Dunn’s post hoc test was applied as appropriate. For transcriptomic analyses, including differential expression and gene set enrichment analyses, *p* values were adjusted using the Benjamini–Hochberg false discovery rate (FDR) method. Effect sizes (Cohen’s d) and 95% confidence intervals were reported where applicable. All tests were two-sided, and *p* < 0.05 was considered statistically significant.

## Results

### Integrated transcriptomic profiling reveals fibroblasts as a central signaling source in the AAA Niche

**Fig. 1:**
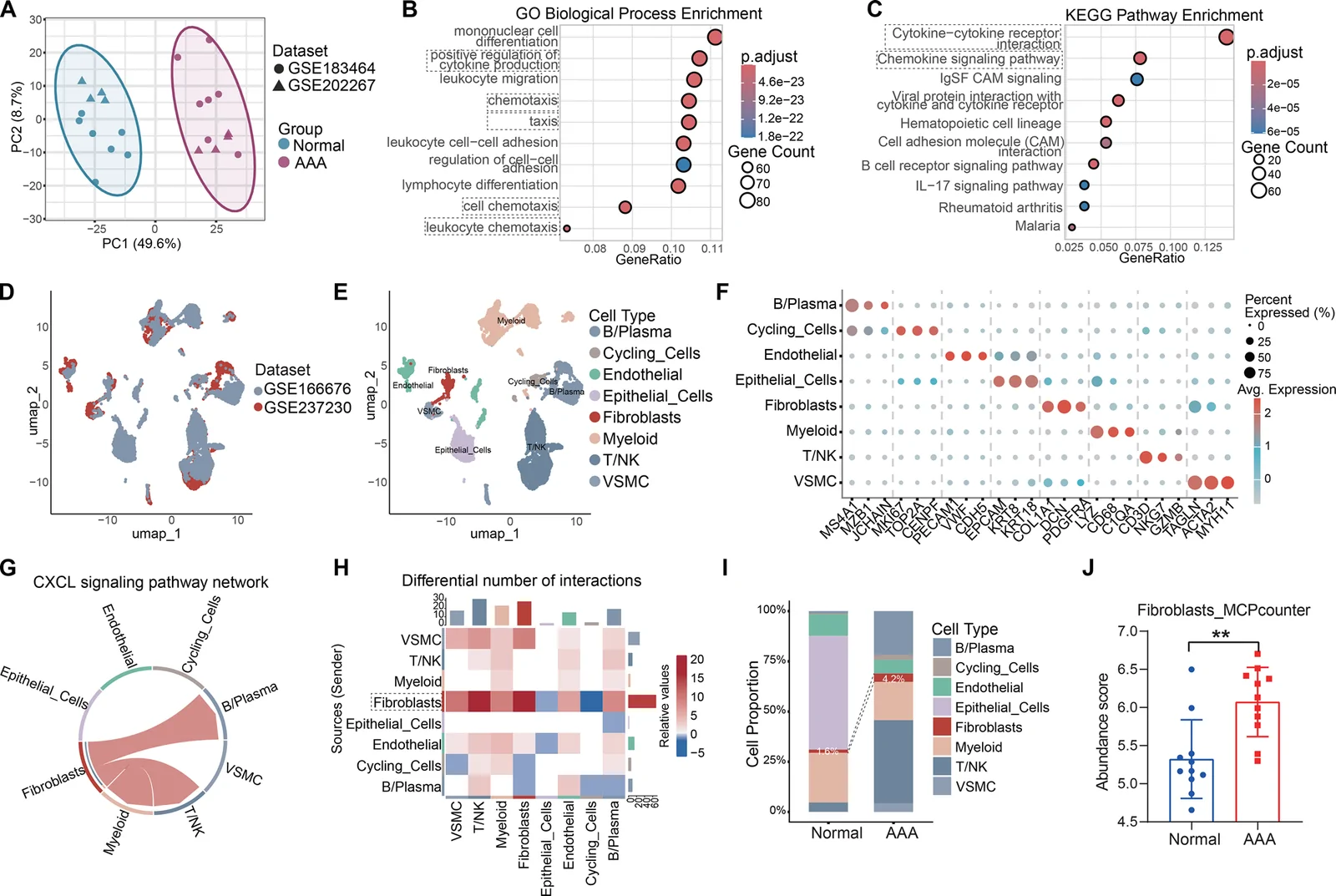
Integrated transcriptomic profiling reveals fibroblasts as a central signaling source in the AAA niche (**A**) Principal component analysis (PCA) of differentially expressed genes from integrated bulk RNA-seq data after ComBat batch-effect correction. Red dots represent AAA samples (*n* = 11); blue dots represent non-aneurysmal controls (*n* = 11). Ellipses denote 95% confidence intervals. (**B**) Bubble plot showing the top 10 enriched gene Ontology biological process (GO-BP) terms based on significantly upregulated genes in AAA (log₂FC > 1, adj.P.Val < 0.05). Dot size and color intensity represent gene count and adjusted *p*-value, respectively. (**C**) Bubble plot displaying the top 10 enriched Kyoto Encyclopedia of genes and Genomes (KEGG) pathways using the same gene set as in (**B**). Dot size and color scale indicate gene number and significance level, respectively. (**D**) UMAP visualization of merged human AAA scRNA-seq datasets from GSE166676 and GSE237230. (**E**) UMAP showing major vascular cell populations identified by canonical marker-based annotation. (**F**) Bubble plot showing average expression of three representative differentially expressed genes (DEGs) across annotated cell type. (**G**) CellChat analysis quantifying outgoing CXCL signaling from each cell type; fibroblasts are the dominant signal source. (**H**) Heatmap of overall outgoing signaling strength per cell type, ranking fibroblasts as the most active signal senders. (**I**) The proportion distribution of all celltypes (% of all nucleated cells) in normal vs. AAA samples. Two-group comparisons used Welch’s two-tailed t-test. (**J**) MCP-counter–based deconvolution of fibroblast abundance in AAA (*n* = 11) and non-aneurysmal control tissues (*n* = 11) using integrated bulk RNA-seq data (GSE183464 and GSE202267). **, *p* < 0.01 (Welch’s two-tailed t-test)

To establish a robust, foundational molecular profile of AAA, we integrated and analyzed bulk RNA-sequencing data from two independent public cohorts (GSE183464 & GSE202267), forming a combined dataset of abdominal aortic tissues from 11 AAA patients and 11 non-aneurysmal controls. Principal component analysis (PCA) based on differentially expressed genes clearly segregated AAA tissues from control samples (Fig. 1A). To define the functional programs underlying AAA progression, Gene Ontology (GO) enrichment analysis was performed on significantly upregulated DEGs. Strikingly, the AAA transcriptome was dominated by chemotaxis-associated inflammatory programs, including “chemotaxis”, “cell chemotaxis”, and “leukocyte chemotaxis” (Fig. 1B). Consistently, Kyoto Encyclopedia of Genes and Genomes (KEGG) analysis confirmed robust activation of the “Chemokine signaling pathway” and “Cytokine-cytokine receptor interaction” (Fig. 1C). Together, these findings reveal a chemokine-centered inflammatory niche within AAA tissues that may drive persistent immune cell recruitment.

Having defined these dominant pathway alterations at the tissue level, we next sought to resolve their cellular origins. To this end, we integrated single-cell RNA sequencing (scRNA-seq) data from 10 human aortic samples (obtained from patients undergoing open AAA repair or aortobifemoral bypass; GSE166676 and GSE237230) Following stringent quality control, UMAP visualization showed no obvious batch-driven clustering across datasets (Supplementary Fig. S1A; Fig. 1D). We constructed a unified cellular atlas of eight major vascular cell types defined by established marker genes and distinct transcriptional signatures (Fig. 1E, F).

Systematic analysis of cell-cell communication (CellChat) revealed that fibroblasts are the dominant source of CXCL signals within AAA lesions, whereas no detectable CXCL signaling was observed in normal aortic tissues (Fig. 1G; Supplementary Fig. S1B–C), and they occupy a leading role as senders across the global intercellular communication network (Fig. 1H). This prominent signaling role coincides with an increased proportion of fibroblasts in AAA as observed in scRNA-seq data (Fig. 1I). To validate this finding in a larger cohort, we performed deconvolution analysis (MCP-counter method) on the aforementioned bulk RNA-seq data, which confirmed the elevated abundance of fibroblasts in AAA tissues (Fig. 1J). In addition, canonical fibroblast markers were more highly expressed in AAA tissues than in controls (Supplementary Fig. S1D).

Taken together, these findings indicate that in AAA, fibroblasts are not passive bystanders but active architects and drivers of the chronic inflammatory microenvironment, positioning them as a prominent signaling source within the AAA microenvironment.

### Single‑Cell profiling reveals Stage‑Specific enrichment of a Rupture‑Associated fibroblast subpopulation

Given the dominant role of fibroblasts as a central signaling hub within the AAA pathological niche, we next sought to dissect their intrinsic functional heterogeneity and to evaluate whether specific fibroblast subpopulations are selectively linked to aneurysm rupture risk.

**Fig. 2:**
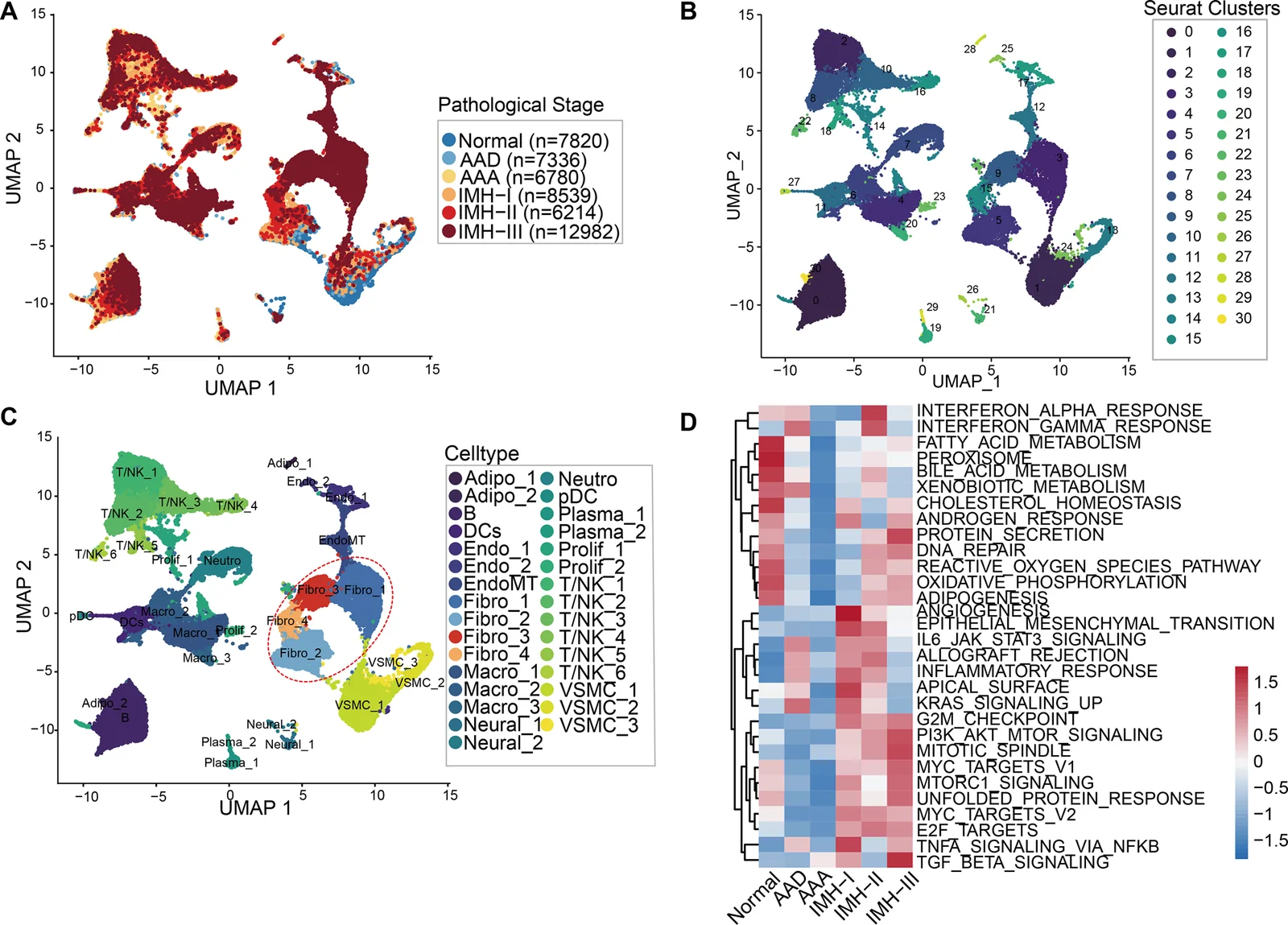
Single-cell transcriptomic profiling of fibroblasts across abdominal aortic aneurysm progression. (**A**) UMAP visualization of 31 transcriptionally distinct subclusters (clustering resolution = 0.1). (**B**) UMAP projection of cells colored by pathological stage. (**C**) UMAP plot annotating refined cell types based on established cell‑specific marker genes. (**D**) Heatmap showing gene set variation analysis (GSVA) scores of fibroblasts across disease stages, based on mouse-specific gene sets from the hallmark (H) and canonical pathways (C2) collections of the molecular signatures Database (MSigDB). Abbreviations: normal, normal aorta; AAD, abdominal aortic dilatation without dissection; AAA, single aortic aneurysm without dissection; IMH-I, aortic dissection/intramural haematoma without aneurysm; IMH-II, single aortic aneurysm with dissection/intramural haematoma; IMH-III, multiple distinct aortic aneurysms with dissection/intramural haematoma

Although existing human AAA single‑cell RNA sequencing datasets have offered valuable insights into cellular composition under steady‑state conditions, their limited scale and general lack of pathological annotations for terminal events such as aneurysm rupture have hindered the systematic reconstruction of cellular dynamics driving rupture progression. To address these limitations, we integrated a single‑cell transcriptomic dataset from a mouse AAA model spanning the full disease spectrum (Fig. 2A, GSE239620). This model adopts the pathological classification system by Daugherty et al. [41, 42], covering a continuum from normal aorta and abdominal aortic dilatation without dissection (AAD), to a single aortic aneurysm without dissection (AAA), and further to increasingly complex stages of dissection/intramural hematoma: IMH‑I (aortic dissection/intramural hematoma without aneurysm), IMH‑II (single aortic aneurysm with dissection/intramural hematoma), and IMH‑III (multiple distinct aortic aneurysms with dissection/intramural hematoma), which corresponds to the most rupture-enriched terminal pathological state. In cross‑section, the medial lumen area remained intact in AAD and AAA regions, whereas the IMH region exhibited full‑thickness medial rupture, resulting in pronounced lumen enlargement and the formation of an intraluminal thrombus infiltrated by immune cells.

Analysis of approximately 50,000 high-quality single-cell transcriptomes initially identified 31 transcriptionally stable clusters at the global level (Fig. 2B). Based on marker‑gene expression profiles, we refined the clustering further to annotate immune cells and four functionally distinct fibroblast subpopulations (Fig. 2C). Cluster stability across a range of resolutions was confirmed using Clustree analysis (Supplementary Fig. S2A). Relative proportions of these subpopulations underwent marked dynamic shifts along the disease continuum, indicating that AAA progression is accompanied by active, stage‑specific remodeling of fibroblast states (Supplementary Fig. S2B-C).

To systematically characterize this functional remodeling at the pathway level, we performed gene‑set variation analysis (GSVA) on fibroblasts isolated from each pathological stage. The results revealed a clear trajectory of metabolic and functional reprogramming. Compared with the normal state, fibroblasts in early disease stages showed suppression of fundamental metabolic pathways together with robust activation of inflammatory signaling. As the disease progressed into late‑stage and rupture‑prone phases, programs associated with fibrotic remodeling—such as epithelial‑mesenchymal transition and TGF‑β signaling—were specifically upregulated (Fig. 2D, Table S2). Notably, in contrast to the inflammation‑dominant signature observed in whole‑tissue analyses, fibroblasts in late‑stage disease displayed a distinct shift toward a pro‑fibrotic phenotype.

Together, these results suggest that AAA progression is accompanied not by uniform fibroblast accumulation, but by stage-specific remodeling of fibroblast states, coinciding with transcriptomic features associated with rupture-enriched lesions. This observation lays the groundwork for subsequent molecular characterization and mechanistic investigation of fibroblast subpopulations associated with aneurysm rupture.

### Functional profiling identifies a pro-rupture COL10A1^+^ fibroblast subpopulation

**Fig. 3:**
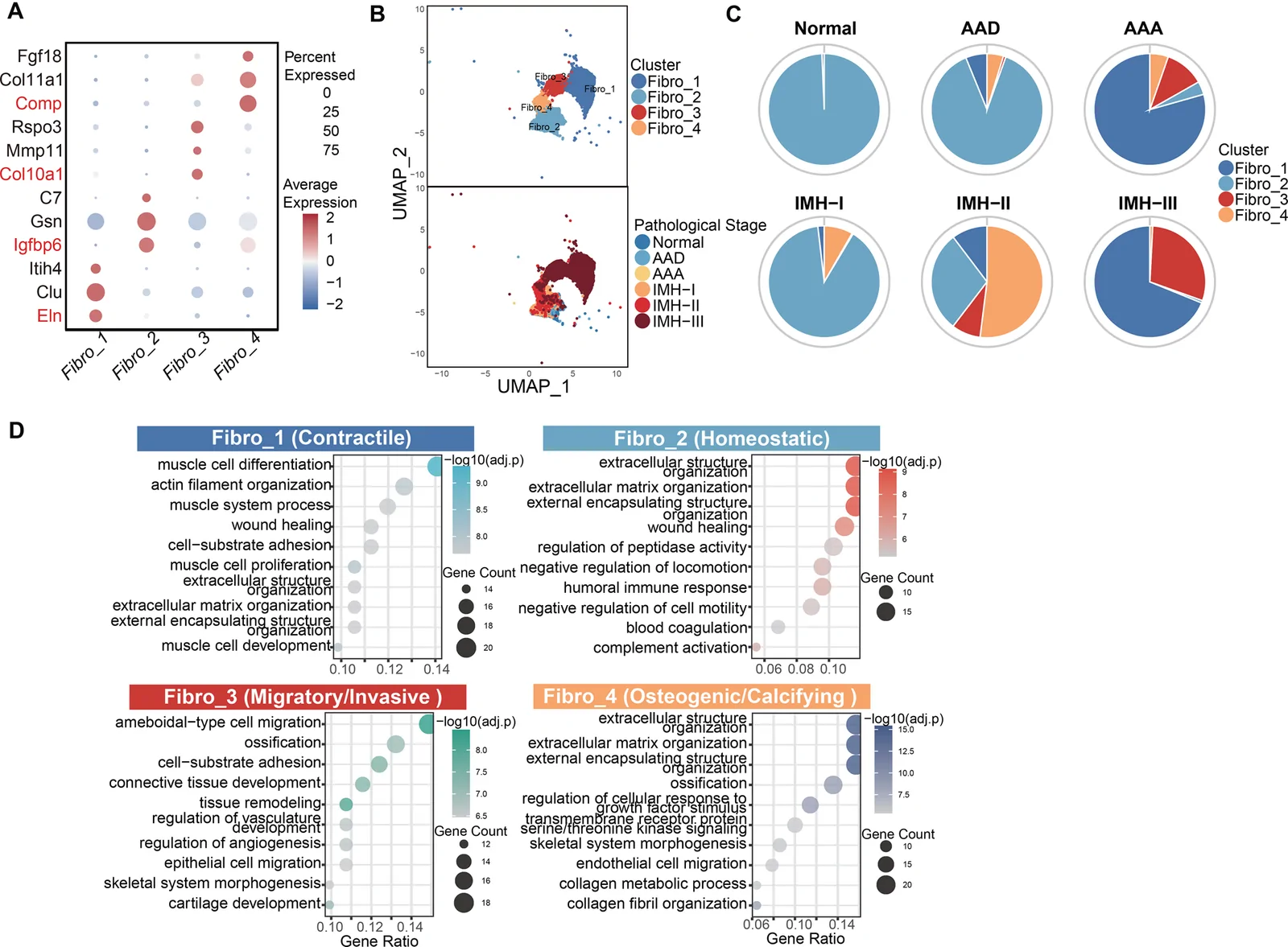
Transcriptional profiling reveals four fibroblast subpopulations with distinct dynamics and functional programs (**A**) Bubble plot of representative marker genes defining each fibroblast subcluster (min.Pct = 0.25, log₂FC > 0.25). (**B**) UMAP visualization of fibroblast subpopulations across pathological stages. (**C**) Pie chart showing fibroblast subpopulation proportions across pathological stages. (**D**) Bubble plot showing the top 10 GO biological processes for each subcluster. Enrichment was performed using the top 150 subcluster-specific marker genes (adjusted *p* value < 0.05, log₂FC > 1)

We characterized the four fibroblast subtypes through transcriptional and functional profiling. Each subtype exhibited distinct marker genes (Fig. 3A) and showed dynamic redistribution across disease stages in UMAP visualization (Fig. 3B). Quantitative analysis revealed pronounced and stage‑specific shifts in subtype proportions (Fig. 3C, Supplementary Fig. S3A): Fibro_1(ELN^+^, matrix-stabilizing) was present during early aneurysmal dilation (AAD, 6.2%) and became the most abundant subtype in rupture‑prone IMH‑III lesions (68.8%); Fibro_2 (IGFBP6^+^, homeostatic) was highly enriched in normal aorta (99.3%) and early non‑aneurysmal stages (AAD: 88.4%; IMH‑I: 89.6%) but nearly absent in IMH‑III (0.7%); Fibro_4 (COMP^+^, fibrotic/osteogenic) was most prominent in IMH‑II lesions (52.0%). Notably, although Fibro_1 constituted the predominant population in IMH‑III, its functional profile was primarily associated with structural maintenance and injury repair. In contrast, Fibro_3 (COL10A1^+^) was virtually undetectable in normal aorta and early, non‑rupture‑prone stages (AAD: 0.7%; IMH‑I: 0.3%), but expanded selectively in aneurysm‑containing stages, rising from 11.5% in AAA to 29.6% in rupture‑prone IMH‑III lesions. This expansion pattern indicates that the increase of Fibro_3 is specifically driven by AAA pathogenesis and correlates strongly with the transition to a rupture‑prone state.

Gene Ontology analysis further delineated subtype‑specific functional programs (Fig. 3D, Supplementary Fig. S3B). Fibro_1 was associated with wound healing, extracellular matrix stabilization and tissue injury repair, reflecting a protective phenotype aimed at limiting damage and maintaining vascular integrity in rupture-prone lesions; Fibro_2 with extracellular matrix organization and immunomodulation; Fibro_4 with collagen fibril organization and osteogenic signaling. Critically, Fibro_3 was uniquely enriched for ameboidal‑type cell migration, together with processes related to active tissue remodeling, cell–matrix adhesion, cartilage development, and angiogenesis regulation—a functional signature that points directly to active vascular wall disruption and destabilization.

Collectively, these findings identify Fibro_3 as a distinct COL10A1^+^ fibroblast state characterized by enhanced migratory and tissue-remodeling activity. Despite being numerically less abundant than Fibro_1 in late-stage lesions, Fibro_3 exhibited a unique functional program closely associated with pathological remodeling and rupture-associated AAA progression.

### Trajectory inference and regulatory network analysis reveal a pathogenic transition toward the COL10A1^+^ fibroblast state

**Fig. 4:**
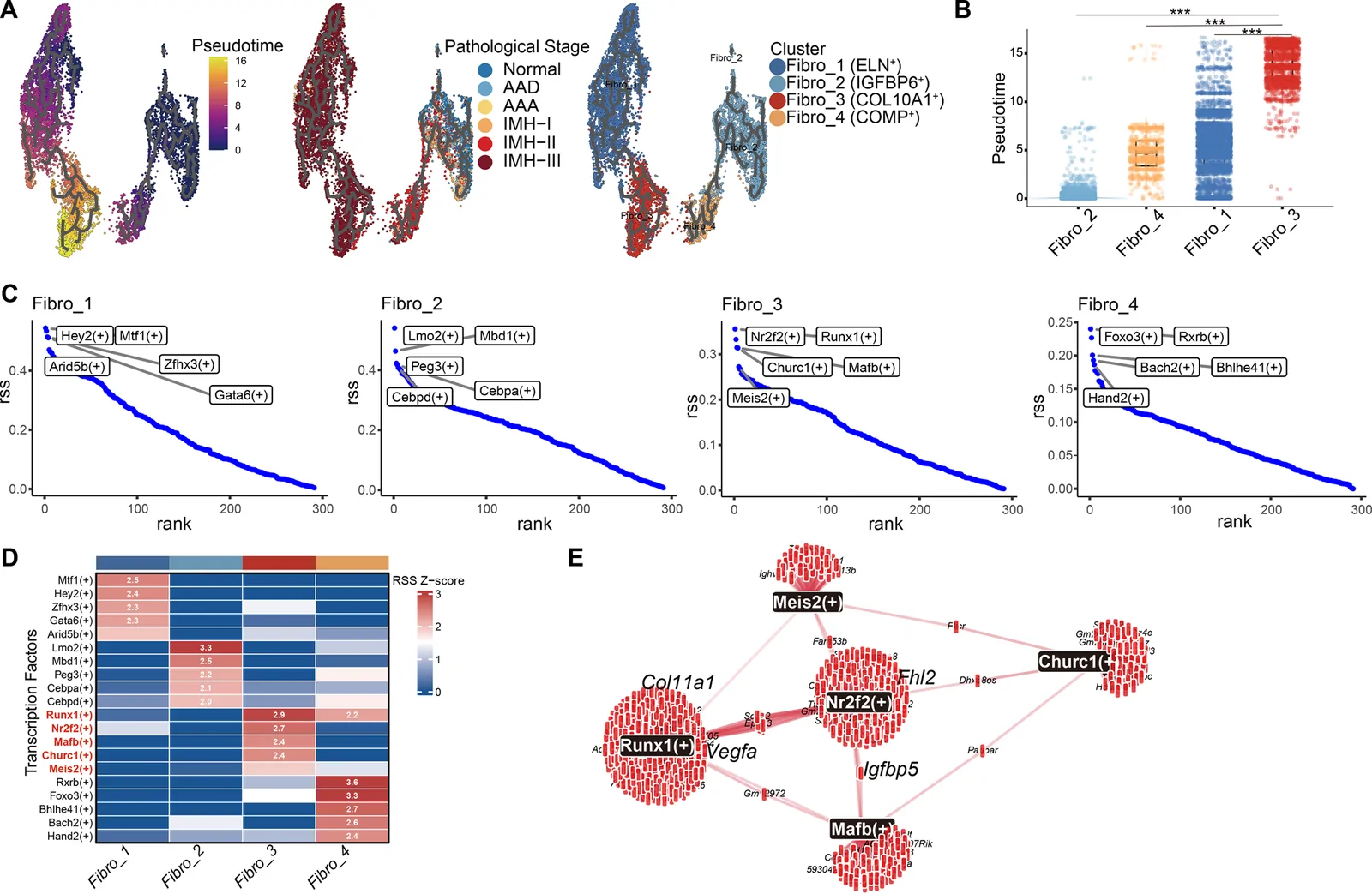
Pseudotime trajectories and regulon analysis of fibroblast subpopulations in AAA. (**A**) Pseudotime trajectory projected onto the low-dimensional manifold. Cells are colored by pseudotime values (left), pathology type (middle), or fibroblast subcluster identity (right). (**B**) Distribution of pseudotime values across fibroblast subclusters, ordered by increasing median pseudotime. Pairwise comparisons between Fibro_3 and other subclusters were performed using two-sided Wilcoxon rank-sum tests. ***, *p* < 0.001. (**C**) Dot plot ranking the top 5 differentially activated regulons in each fibroblast subtype based on regulon-specific scores (RSS). (**D**) Heatmap showing the top 5 regulons across fibroblast subtypes according to RSS Z-score. (**E**) Network of downstream target genes of Fibro_3-specific regulons

To delineate the lineage relationships and progressive transitions among fibroblast subpopulations during abdominal aortic aneurysm progression, we performed pseudotime trajectory analysis using Monocle 3. Fibroblasts were ordered along a continuous developmental trajectory originating from the homeostatic and immunomodulatory Fibro_2 (IGFBP6^+^) cluster and extending toward the terminal Fibro_3 (COL10A1^+^) state (Fig. 4A). Correspondingly, pseudotime scores increased progressively across the four subpopulations, with the lowest values in Fibro_2 and the highest in Fibro_3 (Fig. 4B). The inferred trajectory strongly correlated with disease severity: cells at early pseudotime points were predominantly derived from normal aorta or early-stage aneurysmal dilation, whereas cells at the distal end were almost exclusively sourced from high-risk intramural hematoma type III (IMH-III) lesions. These findings indicate that Fibro_3 is unlikely to represent a pre-existing fibroblast lineage, but instead reflects an acquired disease-associated transcriptional state, induced by sustained pathological stress within the aneurysmal aortic wall.

To investigate transcriptional programs underlying the establishment and maintenance of this pathogenic state, we applied the SCENIC framework to infer cell-type–specific regulons (see Methods). The top 5 regulons per fibroblast subtype were visualized based on regulon-specific scores (RSS, Fig. 4C) and as integrated heatmaps (Fig. 4D). Fibro_3 (COL10A1^+^) exhibited highly specific regulon activity, with Runx1, Nr2f2, Mafb, Churc1, and Meis2 among the top 5, defining the core regulatory network of this pathogenic state.

Analysis of downstream target genes of these Fibro_3 (COL10A1^+^)-specific transcription factors revealed a coordinated effector program associated with disruption of vascular wall integrity (Fig. 4E). Runx1 regulons were linked to pathological angiogenesis and aberrant extracellular matrix remodeling, exemplified by regulation of *Vegfa* and the cartilage/bone-related matrix gene *Col11a1*. Nr2f2 preferentially targeted genes involved in matrix stability and proteolytic balance, including *Serpine2*. *Igfbp5* was identified as a shared target of both Mafb and Churc1 regulons, suggesting its role as an integrative node that consolidates multiple transcriptional inputs to sustain and amplify the pathogenic phenotype of Fibro_3.

Collectively, trajectory inference and regulatory network analyses indicate that the emergence of the Fibro_3 (COL10A1^+^) fibroblast state represents a progressive, disease-associated transition driven by coordinated activity of multiple transcription factors, converging on effector programs that promote angiogenic imbalance and pathological matrix remodeling, thereby aligning with molecular programs associated with AAA progression and rupture susceptibility.

### COL10A1^+^ fibroblasts orchestrate a rupture-prone microenvironment via dynamic ligand–receptor axes

**Fig. 5:**
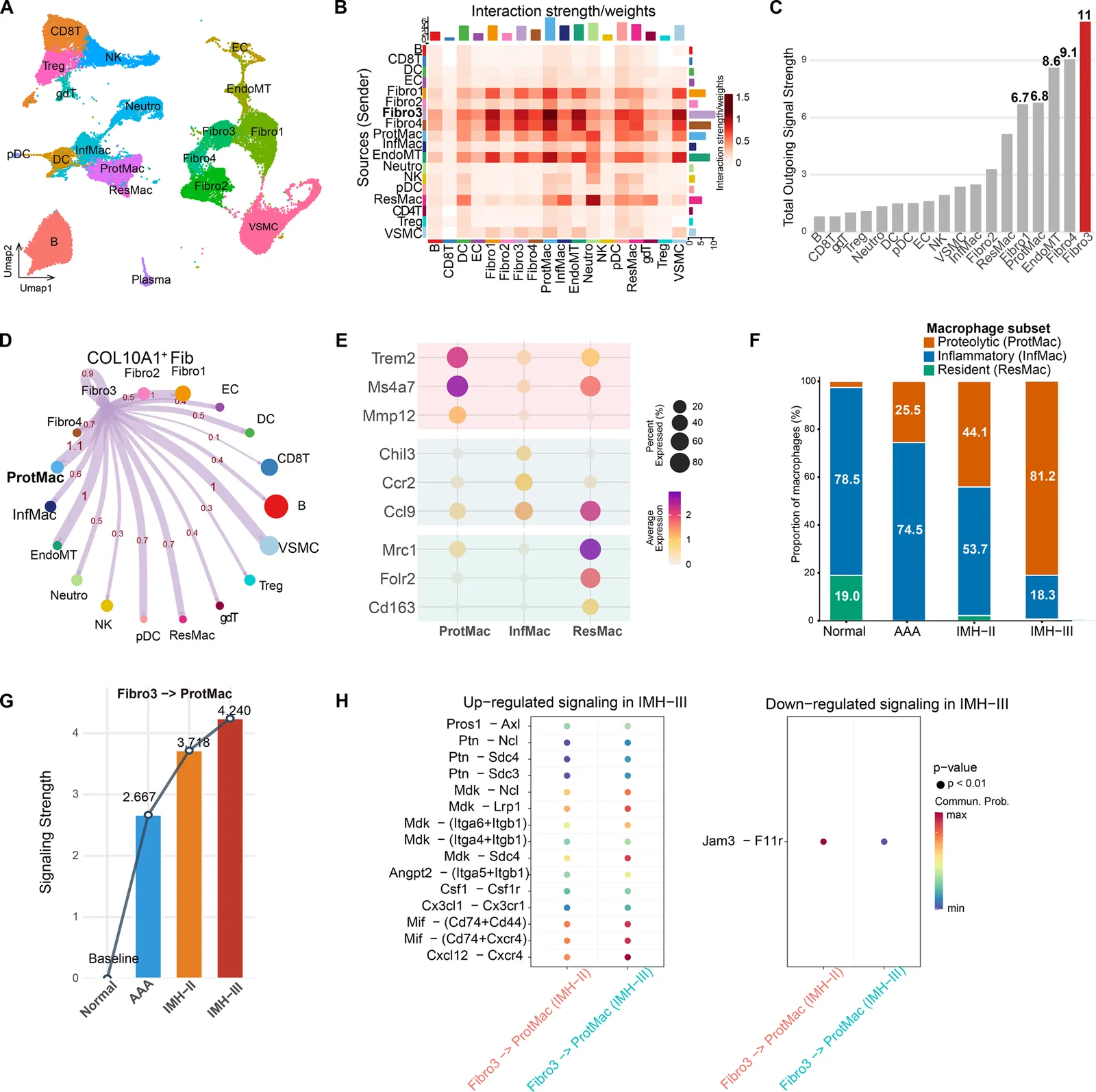
COL10A1^+^ fibroblasts orchestrate a rupture-prone microenvironment via dynamic cell–cell communication. (**A**) UMAP visualization of major cell populations in the integrated AAA single-cell dataset (*n* ≈ 50,000 cells) after exclusion of neurons and rare cell types. (**B**) Heatmap depicting the communication probability of outgoing signaling for each major cell type across the entire network. (**C**) Bar plot summarizing the total outgoing signaling strength per cell population. (**D**) Circle plots depicting the outgoing communication intensity from the COL10A1^+^ Fibro_3 subpopulation to all other cell types in the microenvironment. Line thickness corresponds to interaction strength. (**E**) Dot plot illustrating the expression patterns of the representative marker genes defining proteolytic (ProtMac), inflammatory (InfMac), and resident macrophage (ResMac) subpopulations. (**F**) Stacked bar plot displaying the proportional changes of macrophage subtypes across all AAA pathological stages. (**G**) Bar plot quantifying the stage-specific communication strength between the COL10A1^+^ Fibro-3 subpopulation and proteolytic macrophages (ProtMac). (**H**) Dot plot of selected ligand–receptor pairs mediating Fibro-3–ProtMac crosstalk, highlighting interactions significantly upregulated (left) or downregulated (right) in IMH-III compared to AAA or IMH-II stages. Abbreviations: B (B cells); CD8T (CD8^+^ T cells); DC (dendritic cells); EC (endothelial cells); EndoMT (endothelial-to-mesenchymal transition cells); Fibro_1–4 (fibroblast subpopulations 1–4); γδT (gamma‑delta T cells); InfMac (inflammatory macrophages); Neutro (neutrophils); NK (natural killer cells); pDC (plasmacytoid dendritic cells); plasma (plasma cells); ProtMac (proteolytic macrophages); ResMac (resident‑like macrophages); Treg (regulatory T cells); VSMC (vascular smooth muscle cells)

To investigate how the COL10A1^+^ fibroblast subpopulation (Fibro_3) reshapes the abdominal aortic aneurysm (AAA) microenvironment, we conducted a systematic analysis of cell–cell communication networks. Prior to analysis, neuronal and rare cell populations were excluded, and major vascular and immune cell types were annotated, including four fibroblast subpopulations, vascular smooth muscle cells (VSMCs), endothelial cells (ECs), immune cells, and a specialized cell state arising from endothelial-to-mesenchymal transition (EndoMT) (Fig. 5A; Supplementary Fig. S4A; Table S3). EndoMT cells [43] co-expressed canonical endothelial markers (*Pecam1*, *Cdh5*) together with mesenchymal and chondrogenic markers (*Col1a1*, *Fn1*, *Sox9*), and were selectively enriched in rupture-prone IMH-III lesions, suggesting their potential contribution to late-stage ectopic calcification.

Given the established role of macrophages in AAA pathogenesis, macrophages were further stratified based on their transcriptional programs into three major subpopulations: proteolytic macrophages (ProtMac), inflammatory macrophages (InfMac), and resident macrophages (ResMac). ProtMac were defined by elevated expression of genes associated with extracellular matrix degradation and active tissue remodeling, InfMac were characterized by enrichment of inflammatory response–related gene signatures, whereas ResMac exhibited transcriptional profiles consistent with tissue residency and homeostatic macrophage functions (Supplementary Fig. S4A).

Quantitative analysis of the global communication network revealed that Fibro_3 exhibited the highest outgoing signaling activity within the AAA microenvironment (Fig. 5B, C; Supplementary Fig. S4B-C). Focusing on fibroblast-specific interactions, Fibro_3 displayed highly selective communication, with its strongest outgoing signals directed toward proteolytic macrophages (ProtMac) (Fig. 5D). ProtMac were characterized by high expression of the extracellular matrix–degrading enzyme *Mmp12* and lipid‑associated genes *Trem2* (Fig. 5E). This molecular signature defines a macrophage subset with potent destructive remodeling capacity: MMP12 directly cleaves vascular elastic and collagen fibers, driving wall weakening [44, 45], while TREM2 [46, 47] marks disease-associated macrophages that orchestrate pathological tissue clearance and inflammation, contributing to plaque destabilization and vascular injury. Notably, ProtMac underwent dramatic expansion during disease progression, increasing from 25.5% of macrophages in AAA to 81.2% in IMH-III lesions (Fig. 5F), indicating strong co-evolution with advanced pathological states.

To dissect the dynamic evolution of the Fibro_3–ProtMac axis, we compared its communication strength across disease stages. This interaction was negligible in normal aorta, gradually strengthened from AAA to IMH-II, and peaked in IMH-III lesions (Fig. 5G). Ligand–receptor pair analysis further revealed stage-specific enhancement of signaling pathways converging on three functional categories: chemotactic and recruitment axes mediating monocyte/macrophage attraction and retention (e.g., Cxcl12–Cxcr4, Mif–(Cd74+Cxcr4)); survival and activation axes maintaining macrophage viability and pro-inflammatory phenotype (e.g., Csf1–Csf1r); and matrix remodeling cooperative axes centered on the midkine (Mdk) pathway (e.g., Mdk–Lrp1, Mdk–Sdc4), which simultaneously drive inflammation, angiogenesis, and matrix degradation. In contrast, stabilizing signals associated with endothelial barrier integrity (Jam3–F11r) were markedly attenuated in late-stage lesions (Fig. 5H). This reciprocal enhancement of pro-degradative signaling and reduction of stabilizing interactions exacerbates microenvironmental instability in advanced AAA. Furthermore, to assess whether Fibro_3 and ProtMac-associated transcriptional programs are detectable in human disease, we projected their marker-gene signatures onto independent human AAA bulk RNA-seq datasets (GSE202267 and GSE183464). Both signatures showed increased enrichment in AAA tissues compared with normal controls (Supplementary Fig. S4D-E).

In summary, COL10A1^+^ Fibro_3 cells act as an active signaling hub rather than passive participants, selectively recruiting, activating, and sustaining the expansion of proteolytic macrophages (ProtMac) via dynamically reinforced ligand–receptor axes, particularly those involving Mdk, Cxcl12, and Csf1. Together, these interactions create a self-amplifying loop of uncontrolled matrix degradation and inflammation, which is most prominently observed in rupture-enriched late-stage lesions.

### Fibroblast-derived molecular programs stratify rupture risk beyond aneurysm diameter

To assess whether the pathogenic fibroblast-driven molecular programs identified at the single-cell level could be captured in human abdominal aortic aneurysm (AAA) tissues and associated with rupture risk, we analyzed an independent clinical validation cohort from the GEO database (GSE57691). This dataset contains genome-wide expression profiles of aortic specimens from 59 individuals, including normal donor aortas (*n* = 10), small AAAs (*n* = 20, mean maximum diameter 54.3 ± 2.3 mm), and large AAAs (*n* = 29, mean maximum diameter 68.4 ± 14.3 mm).

**Fig. 6:**
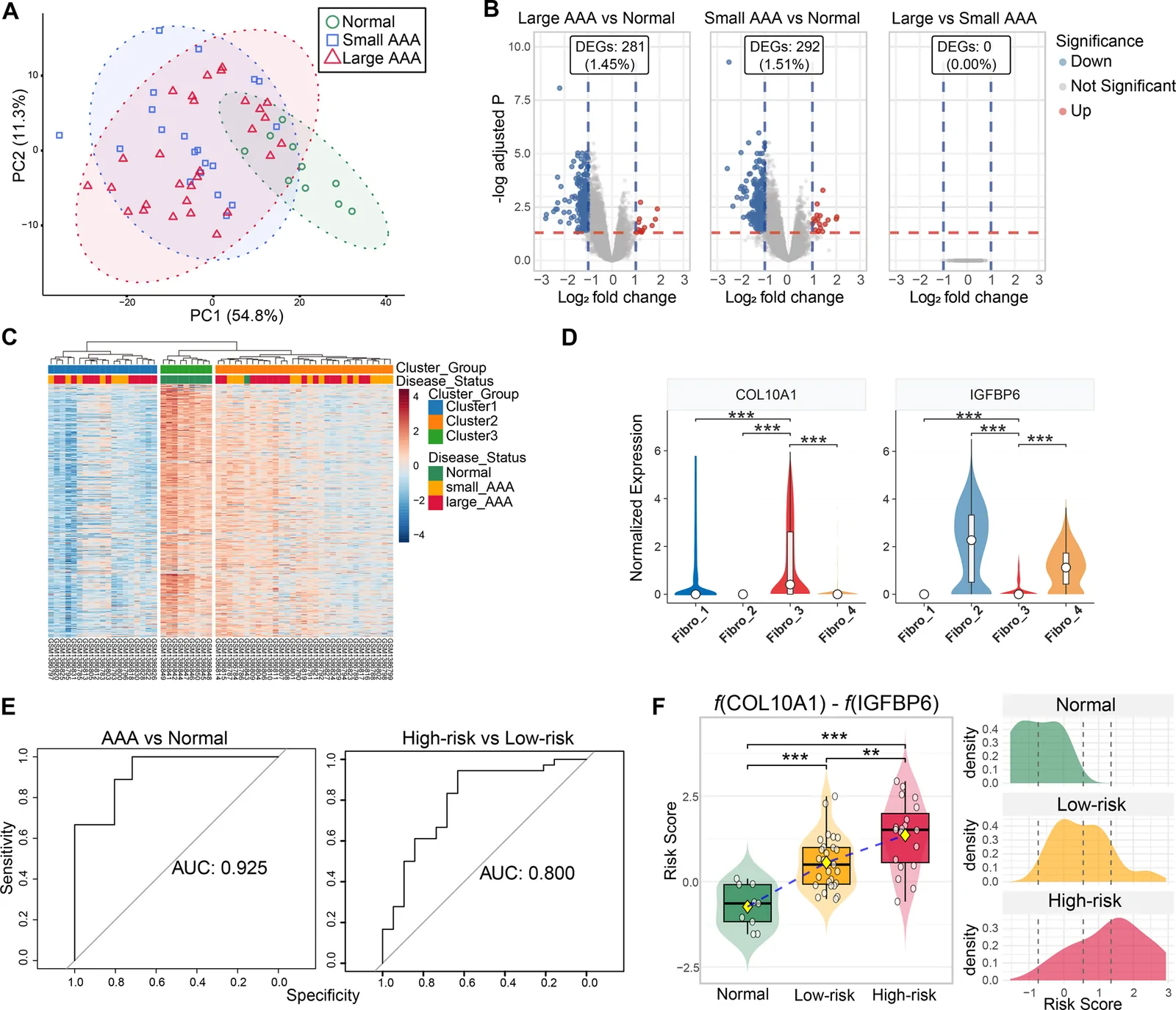
Fibroblast-derived molecular signatures enable rupture risk stratification beyond aneurysm diameter (**A**) Principal component analysis (PCA) of human aortic samples from the GSE57691 cohort based on AAA associated differentially expressed genes. (**B**) Volcano plots depicting differential gene expression. From left to right: large AAA versus normal aorta (281 significant genes), small AAA versus normal aorta (292 significant genes), and large AAA versus small AAA (0 significant genes). Red dots denote significantly differentially expressed genes (adjusted *p*-value < 0.05, FDR correction). (**C**) Unsupervised clustering of AAA samples based on disease-associated transcriptional signatures identifies two distinct molecular subtypes corresponding to low-risk and high-risk AAA. (**D**) Schematic illustration of the fibroblast-derived molecular risk score, defined by the antagonistic expression of the pathogenic Fibro_3 marker gene *COL10A1* and the homeostatic Fibro_2 marker gene *IGFBP6*. (**E**) Receiver operating characteristic (ROC) curves evaluating the performance of the fibroblast-derived risk score in an independent validation cohort. The score shows high accuracy in distinguishing AAA from normal aortic tissues and robust discriminatory power for identifying the high-risk molecular subtype. (**F**) Distribution of the fibroblast-derived risk score across normal, low-risk AAA, and high-risk AAA groups. Violin plots illustrate the overall distribution of scores within each group, while overlaid density plots depict the probability density of risk scores. Statistical comparisons between groups were performed using one-way ANOVA followed by Tukey’s HSD post-hoc test. Significance levels are indicated as: *, *p* < 0.05; **, *p* < 0.01, ***, *p* < 0.001

Unsupervised transcriptomic projection based on AAA-associated differentially expressed genes revealed clear separation between normal aorta samples and all AAA samples, whereas small and large AAAs highly overlapped in transcriptomic space and could not be distinguished from each other (Fig. 6A). Further concordance analysis indicated that, although both small and large AAAs exhibited extensive and significant transcriptional remodeling relative to normal tissues, there were no significantly differentially expressed genes between small and large AAAs (Fig. 6B). These results suggest that maximum aneurysm diameter does not reflect the intrinsic molecular heterogeneity of AAA, and rupture-associated transcriptional heterogeneity is not adequately captured by aneurysm diameter alone.

Based on these findings, we further explored whether fibroblast-dominated pathogenic signals could reveal molecular risk states hidden beneath the diameter phenotype. Unsupervised clustering of AAA samples stratified patients into two molecular subtypes: one with transcriptional profiles highly similar to normal aorta, defined as a low-risk subtype; the other markedly deviating from normal at the transcriptome level, defined as a molecularly high-risk subtype (Fig. 6C). Notably, this molecular stratification was independent of aneurysm diameter, indicating the existence of molecular risk states in AAA that are uncoupled from anatomical size.

To translate these findings into a clinically applicable metric, we developed a minimal fibroblast‑centric molecular risk index. This index integrates two fibroblast markers with opposing functional signatures: the pathogenic driver *COL10A1* (Fibro‑3) and the homeostatic regulator *IGFBP6* (Fibro‑2). The risk score is defined as the arithmetic difference between their standardized expression values (COL10A1 – IGFBP6; Fig. 6D), providing a simple yet biologically grounded measure of the shift from vascular homeostasis to a rupture‑prone state.

In an independent clinical validation cohort, the fibroblast-derived risk score stratified samples along a continuous spectrum from normal aorta, through low-risk AAA, to high-risk AAA. Receiver operating characteristic (ROC) analysis demonstrated excellent accuracy in distinguishing AAA from normal aortic tissues, as well as significant discriminatory power in identifying the high-risk molecular subtype (Fig. 6E). Importantly, this risk score reflects the pathogenic biological state of the aneurysm rather than its anatomical size, highlighting its ability to capture molecular features associated with rupture that are not conveyed by diameter measurements alone (Fig. 6F).

In summary, our study demonstrates that pathogenic COL10A1^+^ fibroblast programs leave a detectable molecular imprint in human AAA tissues and can be distilled into a concise and biologically grounded risk scoring system. This fibroblast-centric molecular signature identifies a subset of “molecularly high-risk” aneurysms that cannot be distinguished by conventional diameter-based criteria, thereby providing a biologically informed framework for molecular risk stratification that complements, rather than replaces, conventional morphology-based assessment.

## Discussion

The progression of aneurysmal disease ultimately culminates in aortic rupture, a catastrophic event whose underlying cellular mechanisms are insufficiently captured by current anatomy-based risk parameters. Here, we demonstrate that AAA progression is associated with an actively regulated pathological program involving specialized cellular states. Through integrated cross-species single-cell analyses, we identify a COL10A1^+^ fibroblast–associated state that emerges selectively during late-stage disease and is enriched within a vascular microenvironment prone to rupture. To our knowledge, this study represents the first to systematically map fibroblast heterogeneity across the full spectrum of AAA progression and to link a specific fibroblast state to biological features associated with rupture susceptibility.

The current clinical paradigm for assessing AAA rupture risk relies predominantly on maximum aortic diameter, which incompletely reflects the biological heterogeneity of the diseased aortic wall [4, 48]. Our findings indicate that disease-associated fibroblast states may provide additional biological information beyond anatomical measurements alone. Within the heterogeneous fibroblast compartment, we identified multiple transcriptionally distinct subtypes with stage‑specific roles in aneurysm pathogenesis: ELN, a major component of elastic fibers critical for vascular integrity [49]; IGFBP6 is associated with the maintenance of tissue homeostasis [50]; COMP drives collagen fibrillogenesis and is linked to fibrotic-osteogenic reprogramming [51]; and COL10A1 is a hallmark of hypertrophic differentiation and pathological tissue remodeling [12, 13].

Although ELN^+^ Fibro_1 constituted the numerically dominant fibroblast population in rupture-prone IMH‑III lesions (69%, Supplementary Fig. S2B), its functional signature—enriched for extracellular matrix stabilization, tissue injury repair, and wound-healing pathways—suggests a reactive, protective role in attempting to limit damage and maintain vascular integrity, rather than an active driver of destabilization (Fig. 3D). This interpretation is further supported by pseudotime trajectory analysis, which positioned Eln^+^ Fibro_1 at a relatively lower differentiation state compared with COL10A1^+^ Fibro_3, indicating a less specialized pathological phenotype (Fig. 4B).

In stark contrast, COL10A1^+^ Fibro_3 displayed defining characteristics of a pathogenic effector population: it emerged specifically during aneurysm progression, expanded dramatically in rupture-prone stages (increasing from 11% in AAA to 30% in IMH‑III), and exhibited a unique functional repertoire centered on ameboid-like migration, aggressive matrix remodeling, and dysregulated angiogenesis. The convergence of stage specificity and pro-remodeling activity positions this fibroblast state as a cellular link between molecular pathology and compromised aortic wall integrity.

The pathogenic influence of COL10A1^+^ Fibro_3 extends beyond autonomous cellular behavior to encompass microenvironmental reprogramming. Through enhanced expression of chemotactic and survival factors, this fibroblast subpopulation establishes a self-reinforcing circuit with proteolytic macrophages, forming a feed-forward signaling network associated with enhanced matrix degradation and inflammatory activity (Fig. 5D–H). This fibroblast–macrophage axis complements established macrophage-centric inflammatory models of AAA by highlighting the contribution of stromal–immune cooperation in sustaining tissue-destructive programs during late-stage disease.

Developmental trajectory analysis revealed that COL10A1^+^ Fibro_3 originates from homeostatic IGFBP6^+^ Fibro_2 fibroblasts through a process of pathological differentiation driven by chronic mechanical and inflammatory stress. The transcriptional regulators associated with this transition—including Runx1, Nr2f2, and Mafb—collectively govern programs related to angiogenic imbalance and pathological matrix remodeling, underscoring that AAA progression involves qualitative shifts in cellular identity in addition to quantitative changes in cell abundance. This highlights that AAA progression involves not merely changes in cell numbers, but fundamental alterations in cellular identity and function.

**Fig. 7:**
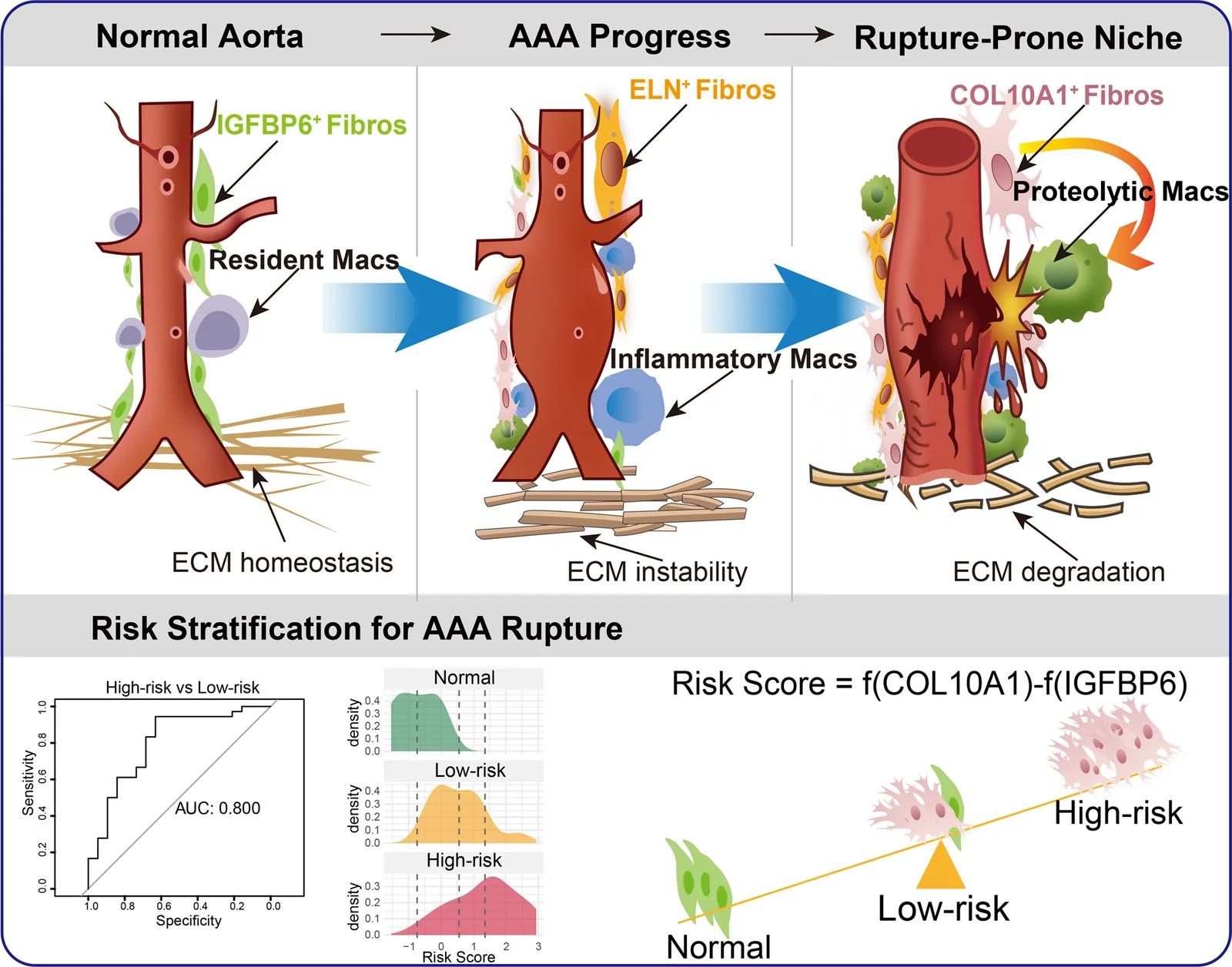
Schematic model of COL10A1^+^ fibroblasts in rupture-prone AAA. IGFBP6^+^ fibroblasts convert into COL10A1^+^ fibroblasts under chronic stress, driving ECM remodeling via interactions with proteolytic macrophages. The dual-gene COL10A1–IGFBP6 risk score translates these single-cell findings into patient stratification beyond aneurysm diameter

Translational implications of this work extend to both risk stratification and therapeutic targeting. A parsimonious dual-gene signature integrating *COL10A1* and *IGFBP6* expression provided improved discrimination of molecular risk states compared with diameter-based assessment alone, supporting its potential utility for molecular risk stratification beyond conventional anatomical assessment. Importantly, this study proposes a fibroblast-derived molecular signature for AAA risk stratification that operates independently of aneurysm diameter (Fig. 7).

Several limitations should be acknowledged. First, the integrated human scRNA-seq dataset contained substantially fewer control samples than AAA samples because of the limited availability of publicly accessible normal human abdominal aortic scRNA-seq datasets. To reduce the potential impact of this imbalance, we performed independent validation in a well-balanced bulk RNA-seq cohort, which yielded consistent fibroblast-associated findings. Importantly, the major conclusions of this study are centered on fibroblast heterogeneity, state transitions, and intercellular communication remodeling rather than differences in overall fibroblast abundance. Nevertheless, validation in larger human scRNA-seq cohorts will be necessary to further confirm these observations.

Second, although cross-species integration enhances biological inference, validation in well-annotated human AAA specimens across defined stages of rupture risk will be essential. Our functional predictions, though supported by pathway analysis and ligand‑receptor network modeling, require experimental validation through targeted perturbation of COL10A1^+^ Fibro_3 in preclinical models. Furthermore, the complex multicellular ecosystem of the aneurysmal aorta—encompassing endothelial cells, smooth muscle subpopulations, and diverse immune subsets—undoubtedly contributes additional layers of regulation that future spatial multi‑omics approaches will help to elucidate.

Third, although the COL10A1–IGFBP6 score identified biologically distinct AAA subtypes, the validation cohort did not include clinically confirmed ruptured AAA specimens. Therefore, the current findings support molecular risk stratification and the identification of rupture-associated biological states rather than direct prediction of clinical rupture events. Future studies incorporating longitudinal follow-up and clinically ruptured AAA samples will be required to determine the prognostic utility of this signature.

In summary, this study reconceptualizes AAA rupture not as an inevitable mechanical endpoint, but as a biologically driven process mediated by specialized fibroblast subpopulations. By identifying a COL10A1^+^ fibroblast–associated program enriched in advanced disease states associated with rupture susceptibility, we provide a mechanistic framework for understanding aortic wall destabilization and a molecular foundation for advancing biologically informed risk stratification and precision management of AAA.

## Electronic supplementary material

Below is the link to the electronic supplementary material.

Supplementary material 1

Supplementary material 2

Supplementary material 3

Supplementary material 4

## Data Availability

All transcriptomic datasets analyzed in this study are publicly available from the Gene Expression Omnibus (GEO) repository. Human bulk RNA-seq datasets are available under accession numbers **GSE183464**, **GSE202267**, and **GSE57691**. Human single-cell RNA-seq datasets are available under **GSE166676** and **GSE237230**. Mouse single-cell RNA-seq data covering the full AAA progression continuum are available under **GSE239620**. A summary of all datasets, including sample type, species, disease stage, and sample size, is provided in Supplementary Table S1. All custom analysis scripts used for data processing, analysis, and figure generation are publicly available at https://github.com/DanLiu527/AAA.

## References

[1] Reimerink JJ, van der Laan MJ, Koelemay MJ, Balm R, Legemate DA. Systematic review and meta-analysis of population-based mortality from ruptured abdominal aortic aneurysm. Br J Surg. 2013;100(11):1405–13. 10.1002/bjs.9235. 24037558

[2] Sakalihasan N, Michel JB, Katsargyris A, Kuivaniemi H, Defraigne JO, Nchimi A, et al. Abdominal aortic aneurysms. Nat Rev Disease Primers. 2018;4(1):34. 10.1038/s41572-018-0030-7. 30337540

[3] Wyss TR, Giardini M, Sörelius K, Adam D, Assadian O, Beck AW, et al. Infective native aortic aneurysm: a Delphi consensus document on treatment, follow up, and definition of cure. Eur J Vasc Endovasc Surg. 2024;67(4):654–61. 10.1016/j.ejvs.2023.12.008. 38097164

[4] Vaitėnas G, Mosenko V, Račytė A, Medelis K, Skrebūnas A, Baltrūnas T. Abdominal Aortic Aneurysm Diameter versus Volume: A Systematic Review. 2023. Biomedicines. 2023;11(3):941. 10.3390/biomedicines11030941. PMC10046268 36979920

[5] Kontopodis N, Tzirakis K, Ioannou CV. The obsolete maximum diameter criterion, the evident role of biomechanical (pressure) indices, the new role of hemodynamic (flow) indices, and the multi-modal approach to the rupture risk assessment of abdominal aortic aneurysms. Ann Vasc Dis. 2018;11(1):78–83. 10.3400/avd.ra.17-00115. PMC5882355 29682111

[6] Schahab N, Würbel S, Busch L, Nickenig G. Abdominal aortic aneurysm progression: a review of preclinical and clinical data. Clin Res Cardiol. 2025;115(8):1272–94. 10.1007/s00392-025-02764-8. PMC13346152 41212232

[7] Li H, Bai S, Ao Q, Wang X, Tian X, Li X, et al. Modulation of immune-inflammatory responses in abdominal aortic aneurysm: emerging molecular targets. J Immunol Res. 2018, 2018: 2018:1–15. 10.1155/2018/7213760. PMC6008668 29967801

[8] Gurung R, Choong AM, Woo CC, Foo R, Sorokin V. Genetic and epigenetic mechanisms underlying vascular smooth muscle cell phenotypic modulation in abdominal aortic aneurysm. Int J Mol Sci. 2020;21(17):21. 10.3390/ijms21176334. PMC7504666 32878347

[9] Zou AE, Kongthong S, Mueller AA, Brenner MB. Fibroblasts in immune responses, inflammatory diseases and therapeutic implications. Nat Rev Rheumatol. 2025;21(6):336–54. 10.1038/s41584-025-01259-0. 40369134

[10] Xu Y, Li W, Lin S, Liu B, Wu P, Li L. Fibroblast diversity and plasticity in the tumor microenvironment: roles in immunity and relevant therapies. Cell Commun Signal. 2023;21(1):234. 10.1186/s12964-023-01204-2. PMC10506315 37723510

[11] Yi Q, Zhu G, Zhu W, Wang J, Ouyang X, Yang K, et al. Oncogenic mechanisms of COL10A1 in cancer and clinical challenges (review). Oncol Rep. 2024, 52;52(6). 10.3892/or.2024.8821. PMC11487528 39392043

[12] Kahlert UD, Shi W, Strecker M, Scherpinski LA, Wartmann T, Dölling M, et al. COL10A1 allows stratification of invasiveness of colon cancer and associates to extracellular matrix and immune cell enrichment in the tumor parenchyma. Front Oncol. 2022;12:1007514. 10.3389/fonc.2022.1007514. PMC9577326 36267978

[13] Hu S, Ding M, Lou J, Qin J, Chen Y, Liu Z, et al. COL10A1+ fibroblasts promote colorectal cancer metastasis and M2 macrophage polarization with pan-cancer relevance. J Exp Clin Cancer Res. 2025;44(1):243. 10.1186/s13046-025-03510-8. PMC12360028 40826474

[14] Yang W, Wu X, Zhou F. Collagen type X alpha 1 (COL10A1) contributes to cell proliferation, migration, and invasion by targeting prolyl 4-hydroxylase beta polypeptide (P4HB) in breast cancer. Med Sci Monit. 2020;27:e928919. 10.12659/msm.928919. PMC7927362 33637669

[15] Stenmark KR, Yeager ME, El Kasmi KC, Nozik-Grayck E, Gerasimovskaya EV, Li M, et al. The adventitia: essential regulator of vascular wall structure and function. Annu Rev Physiol. 2013;75(1):23–47. 10.1146/annurev-physiol-030212-183802. PMC3762248 23216413

[16] Mackay CDA, Jadli AS, Fedak PWM, Patel VB. Adventitial fibroblasts in aortic aneurysm: unraveling pathogenic contributions to vascular disease. Diagn (Basel, Switz). 2022;12(4):12. 10.3390/diagnostics12040871. PMC9025866 35453919

[17] Yang H, Zhou T, Stranz A, DeRoo E, Liu B. Single-cell RNA sequencing reveals heterogeneity of vascular cells in early stage murine abdominal aortic aneurysm—brief report. Arterioscler Thromb Vasc Biol. 2021;41(3):1158–66. 10.1161/atvbaha.120.315607. PMC7904588 33472403

[18] Davis FM, Tsoi LC, Melvin WJ, DenDekker A, Wasikowski R, Joshi AD, et al. Inhibition of macrophage histone demethylase JMJD3 protects against abdominal aortic aneurysms. J Exp Med. 2021;218(6):218. 10.1084/jem.20201839. PMC8008365 33779682

[19] Callow B, He X, Juriga N, Mangum KD, Joshi A, Xing X, et al. Inhibition of vascular smooth muscle cell PERK/ATF4 ER stress signaling protects against abdominal aortic aneurysms. JCI Insight. 2025;10(2):10. 10.1172/jci.insight.183959. PMC11790032 39846252

[20] Fasolo F, Winski G, Li Z, Wu Z, Winter H, Ritzer J, et al. The circular RNA ataxia telangiectasia mutated regulates oxidative stress in smooth muscle cells in expanding abdominal aortic aneurysms. Mol Ther - Nucleic Acids. 2023;33:848–65. 10.1016/j.omtn.2023.08.017. PMC10481153 37680984

[21] Zhang K, Yue J, Yin L, Chen J, Chen Y, Hu L, et al. Comprehensive bioinformatics analysis revealed potential key genes and pathways underlying abdominal aortic aneurysm. Comput Struct Biotechnol J. 2023;21:5423–33. 10.1016/j.csbj.2023.10.052. PMC10665597 38022704

[22] Carmona-Berrio D, Adarve-Rengifo I, Marshall AG, Vue Z, Hall DD, Miller-Fleming TW, et al. SOX6 expression and aneurysms of the thoracic and abdominal aorta. iScience. 2024;27(9):110436. 10.1016/j.isci.2024.110436. PMC11388018 39262802

[23] Robinson MD, McCarthy DJ, Smyth GK. edgeR: a Bioconductor package for differential expression analysis of digital gene expression data. Bioinf (Oxford, Engl). 2010;26(1):139–40. 10.1093/bioinformatics/btp616. PMC2796818 19910308

[24] Lawrence M, Huber W, Pagès H, Aboyoun P, Carlson M, Gentleman R, et al. Software for computing and annotating genomic ranges. PLoS Comput Biol. 2013;9(8):e1003118. 10.1371/journal.pcbi.1003118. PMC3738458 23950696

[25] Robinson MD, Oshlack A. A scaling normalization method for differential expression analysis of RNA-seq data. Genome Biol. 2010;11(3):R25. 10.1186/gb-2010-11-3-r25. PMC2864565 20196867

[26] Leek JT, Johnson WE, Parker HS, Jaffe AE, Storey JD. The sva package for removing batch effects and other unwanted variation in high-throughput experiments. Bioinf (Oxford, Engl). 2012;28(6):882–83. 10.1093/bioinformatics/bts034. PMC3307112 22257669

[27] Ritchie ME, Phipson B, Wu D, Hu Y, Law CW, Shi W, et al. Limma powers differential expression analyses for RNA-sequencing and microarray studies. Nucleic Acids Res. 2015;43(7):e47. 10.1093/nar/gkv007. PMC4402510 25605792

[28] Wu T, Hu E, Xu S, Chen M, Guo P, Dai Z, et al. clusterProfiler 4.0: a universal enrichment tool for interpreting omics data. Innov. 2021;2(3):100141. 10.1016/j.xinn.2021.100141. PMC8454663 34557778

[29] Becht E, Giraldo NA, Lacroix L, Buttard B, Elarouci N, Petitprez F, et al. Estimating the population abundance of tissue-infiltrating immune and stromal cell populations using gene expression. Genome Biol. 2016;17(1):218. 10.1186/s13059-016-1070-5. PMC5073889 27765066

[30] Le S, Wu J, Liu H, Du Y, Wang D, Luo J, et al. Single-cell RNA sequencing identifies interferon-inducible monocytes/macrophages as a cellular target for mitigating the progression of abdominal aortic aneurysm and rupture risk. Cardiovasc Res. 2024;120(11):1351–64. 10.1093/cvr/cvae117 %J Cardiovascular Research. 38836630

[31] Hao Y, Hao S, Andersen-Nissen E, Mauck WM 3rd, Zheng S, Butler A, et al. Integrated analysis of multimodal single-cell data. Cell. 2021;184(13):3573–87.e29. 10.1016/j.cell.2021.04.048. PMC8238499 34062119

[32] Zappia L, Oshlack A. Clustering trees: a visualization for evaluating clusterings at multiple resolutions. GigaScience. 2018;7(7):7. 10.1093/gigascience/giy083. PMC6057528 30010766

[33] Hänzelmann S, Castelo R, Guinney J. GSVA: gene set variation analysis for microarray and RNA-seq data. BMC Bioinf. 2013;14(1):7. 10.1186/1471-2105-14-7. PMC3618321 23323831

[34] Liberzon A, Birger C, Thorvaldsdóttir H, Ghandi M, Mesirov JP, Tamayo P. The molecular signatures database hallmark gene set collection. Cell Syst. 2015;1(6):417–25. 10.1016/j.cels.2015.12.004. PMC4707969 26771021

[35] Gu Z, Eils R, Schlesner M. Complex heatmaps reveal patterns and correlations in multidimensional genomic data. Bioinf (Oxford, Engl). 2016;32(18):2847–49. 10.1093/bioinformatics/btw313. 27207943

[36] Cao J, Spielmann M, Qiu X, Huang X, Ibrahim DM, Hill AJ, et al. The single-cell transcriptional landscape of mammalian organogenesis. Nature. 2019;566(7745):496–502. 10.1038/s41586-019-0969-x. PMC6434952 30787437

[37] de Sande B V, Flerin C, Davie K, De Waegeneer M, Hulselmans G, Aibar S, et al. A scalable SCENIC workflow for single-cell gene regulatory network analysis. Nat Protoc. 2020;15(7):2247–76. 10.1038/s41596-020-0336-2. 32561888

[38] Jin S, Guerrero-Juarez CF, Zhang L, Chang I, Ramos R, Kuan CH, et al. Inference and analysis of cell-cell communication using CellChat. Nat Commun. 2021;12(1):1088. 10.1038/s41467-021-21246-9. PMC7889871 33597522

[39] Biros E, Gäbel G, Moran CS, Schreurs C, Lindeman JHN, Walker PJ, et al. Differential gene expression in human abdominal aortic aneurysm and aortic occlusive disease. Oncotarget. 2015;6(15):12984–96. 10.18632/oncotarget.3848. PMC4536993 25944698

[40] DeLong ER, DeLong DM, Clarke-Pearson DL. Comparing the areas under two or more correlated receiver operating characteristic curves: a nonparametric approach. Biometrics. 1988;44(3):837–45. 10.2307/2531595. 3203132

[41] Daugherty A, Manning MW, Cassis LA. Angiotensin II promotes atherosclerotic lesions and aneurysms in apolipoprotein E–deficient mice. J Clin Invest. 2000;105(11):1605–12. 10.1172/jci7818. PMC300846 10841519

[42] Daugherty A, Manning MW, Cassis LA. Antagonism of AT2 receptors augments angiotensin II-induced abdominal aortic aneurysms and atherosclerosis. Br J Pharmacol. 2001;134(4):865–70. 10.1038/sj.bjp.0704331. PMC1573019 11606327

[43] Kenswil KJG, Pisterzi P, Sánchez-Duffhues G, van Dijk C, Lolli A, Knuth C, et al. Endothelium-derived stromal cells contribute to hematopoietic bone marrow niche formation. Cell STEM Cell. 2021;28(4):653–70.e11. 10.1016/j.stem.2021.01.006. 33561425

[44] Di Gregoli K, Atkinson G, Williams H, George SJ, Johnson JL. Pharmacological inhibition of MMP-12 exerts protective effects on angiotensin II-Induced abdominal aortic aneurysms in apolipoprotein E-Deficient mice. Int J Mol Sci. 2024, 25;25(11):5809. 10.3390/ijms25115809. PMC11172660 38891996

[45] Chen Y, Yang X, Kitajima S, Quan L, Wang Y, Zhu M, et al. Macrophage elastase derived from adventitial macrophages modulates aortic remodeling. Front Cell Dev Biol. 2023;10:1097137. 10.3389/fcell.2022.1097137. PMC9871815 36704203

[46] Guo X, Li B, Wen C, Zhang F, Xiang X, Nie L, et al. TREM2 promotes cholesterol uptake and foam cell formation in atherosclerosis. Cell Mol Life Sci. 2023;80(5):137. 10.1007/s00018-023-04786-9. PMC11071710 37133566

[47] Thayaparan D, Emoto T, Khan AB, Besla R, Hamidzada H, El-Maklizi M, et al. Endothelial dysfunction drives atherosclerotic plaque macrophage-dependent abdominal aortic aneurysm formation. Nat Immunol. 2025;26(5):706–21. 10.1038/s41590-025-02132-8. 40263614

[48] Powell JT, Gotensparre SM, Sweeting MJ, Brown LC, Fowkes FGR, Thompson SG. Rupture rates of small abdominal aortic aneurysms: a systematic review of the literature. Eur J Vasc Endovasc Surg. 2011;41(1):2–10. 10.1016/j.ejvs.2010.09.005. 20952216

[49] Lin CJ, Cocciolone AJ, Wagenseil JE. Elastin, arterial mechanics, and stenosis. Am J Physiol-Cell Physiol. 2022;322(5):C875–c886. 10.1152/ajpcell.00448.2021. PMC9037699 35196168

[50] Su M, Zhao W, Jiang H, Zhao Y, Liao Z, Liu Z, et al. Endothelial IGFBP6 suppresses vascular inflammation and atherosclerosis. Nat Cardiovasc Res. 2025;4(2):145–62. 10.1038/s44161-024-00591-0. PMC11825279 39794479

[51] Yin Y, Chen Y, Xu J, Liu B, Zhao Y, Tan X, et al. Molecular and spatial signatures of human and rat corpus cavernosum physiopathological processes at single-cell resolution. Cell Rep. 2024;43(9):114760. 10.1016/j.celrep.2024.114760. 39299236

