# Supplementary material for: COL10A1+ fibroblasts define rupture-prone abdominal aortic aneurysms and enable molecular risk stratification
Source: J Transl Med. 2026 Jul 23;24:1322. doi: 10.1186/s12967-026-08689-8 (PMC13681811; doi:10.1186/s12967-026-08689-8)
Supplement: Supplementary file 1 [file 12967_2026_8689_MOESM1_ESM.pdf]

Supplementary Figures

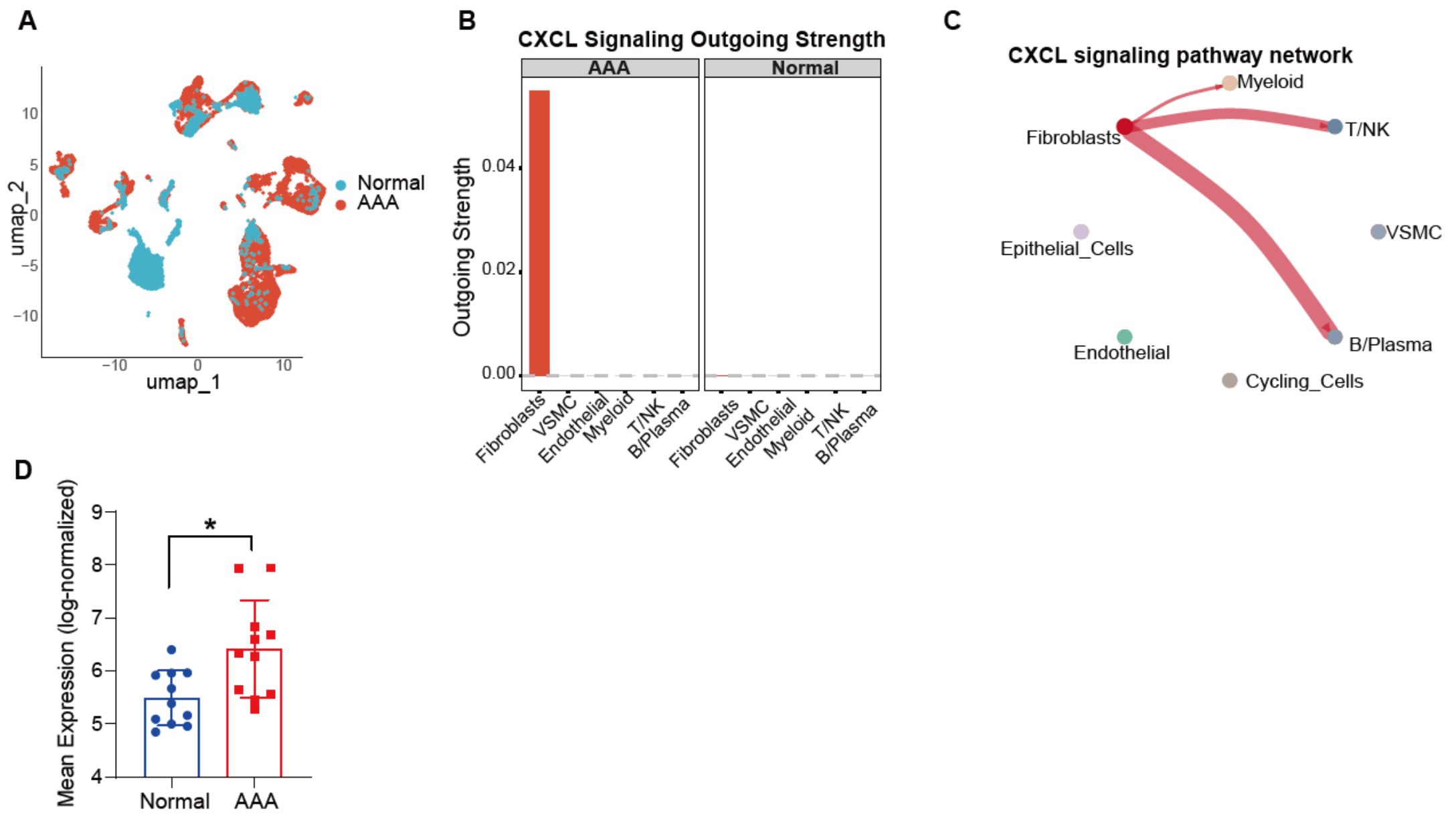

Supplementary Fig. S1. Human aortic scRNA-seq atlas and fibroblast-enriched CXCL signaling in AAA (related to Figure 1).

(A) UMAP of integrated human scRNA-seq datasets from AAA and normal control aortas.

(B) Comparison of CXCL signaling between AAA and normal aortic tissues. Bar plots showing CXCL outgoing signaling strength across all annotated cell types in AAA (left) and normal (right) tissues.

(C) CXCL signaling network in the human AAA microenvironment. Circle plot visualization of CXCL ligand-receptor interactions. Node size represents signaling output strength per cell type; edge width represents interaction strength between cell pairs.

(D) Expression analysis of canonical fibroblast markers in an independent bulk RNA-seq cohort (11 AAA vs. 11 controls). The analyzed marker set includes *COL1A1*, *COL1A2*, *COL3A1*, *DCN*, *LUM*, *PDGFRA*, and *FAP*. *$p < 0.05$ (Welch's t-test).

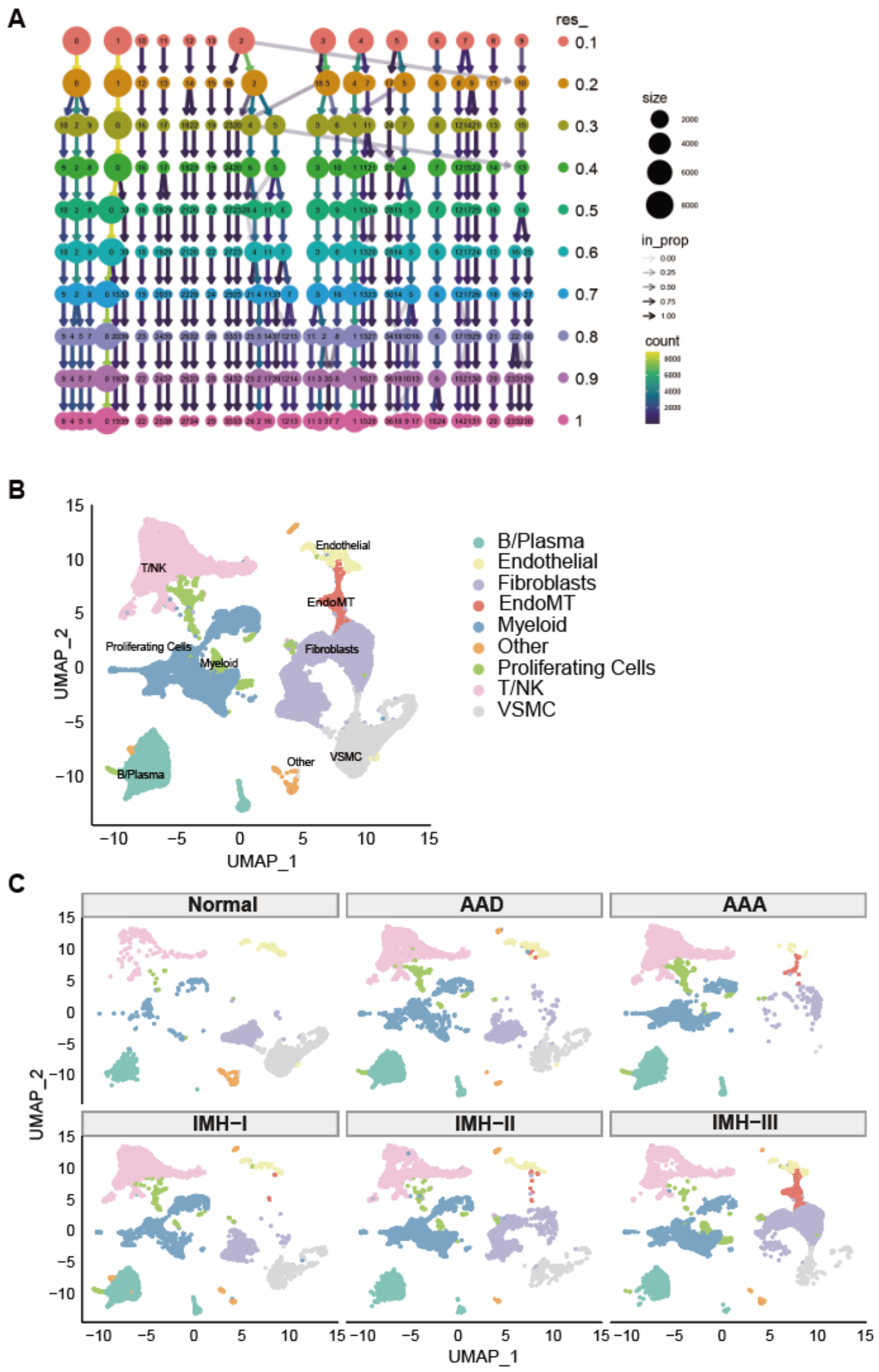

Supplementary Fig. S2. Single-cell atlas of the AAA microenvironment in the mouse model (Related to Figure 2).

(A) Clustree plot showing cluster transitions across Seurat resolutions (0.1–1.0). The four subpopulations remained consistently identifiable across a broad range of resolutions.

(B) UMAP visualization showing annotation of major cell types based on established marker genes.

(C) Stage-wise distribution of annotated cell populations across the AAA disease continuum (UMAP projection).

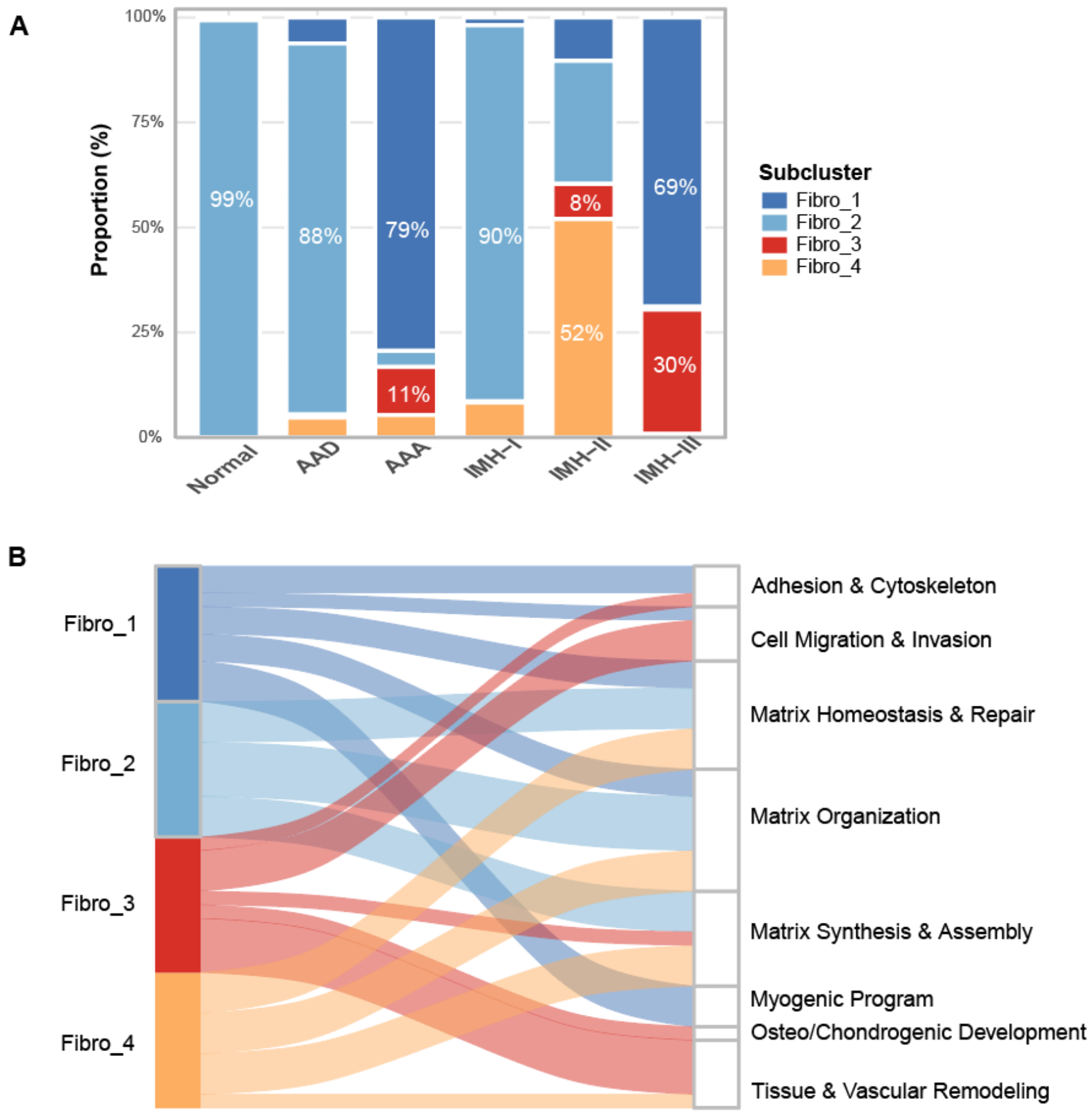

Supplementary Fig. S3. Compositional dynamics and pathway enrichment of fibroblast subpopulations in AAA progression (Related to Figure 3).

(A) Quantification of the relative abundance of the four fibroblast subpopulations (Fibro_1 to Fibro_4) across the pathological continuum (Normal, AAD, AAA, IMH-I, IMH-II, IMH-III), presented as a stacked bar chart.

(B) Sankey diagram illustrating the flow and association between individual fibroblast transcriptional subclusters (source) and their significantly enriched Gene Ontology (GO) biological process terms (target). Line width corresponds to the strength of the enrichment association.

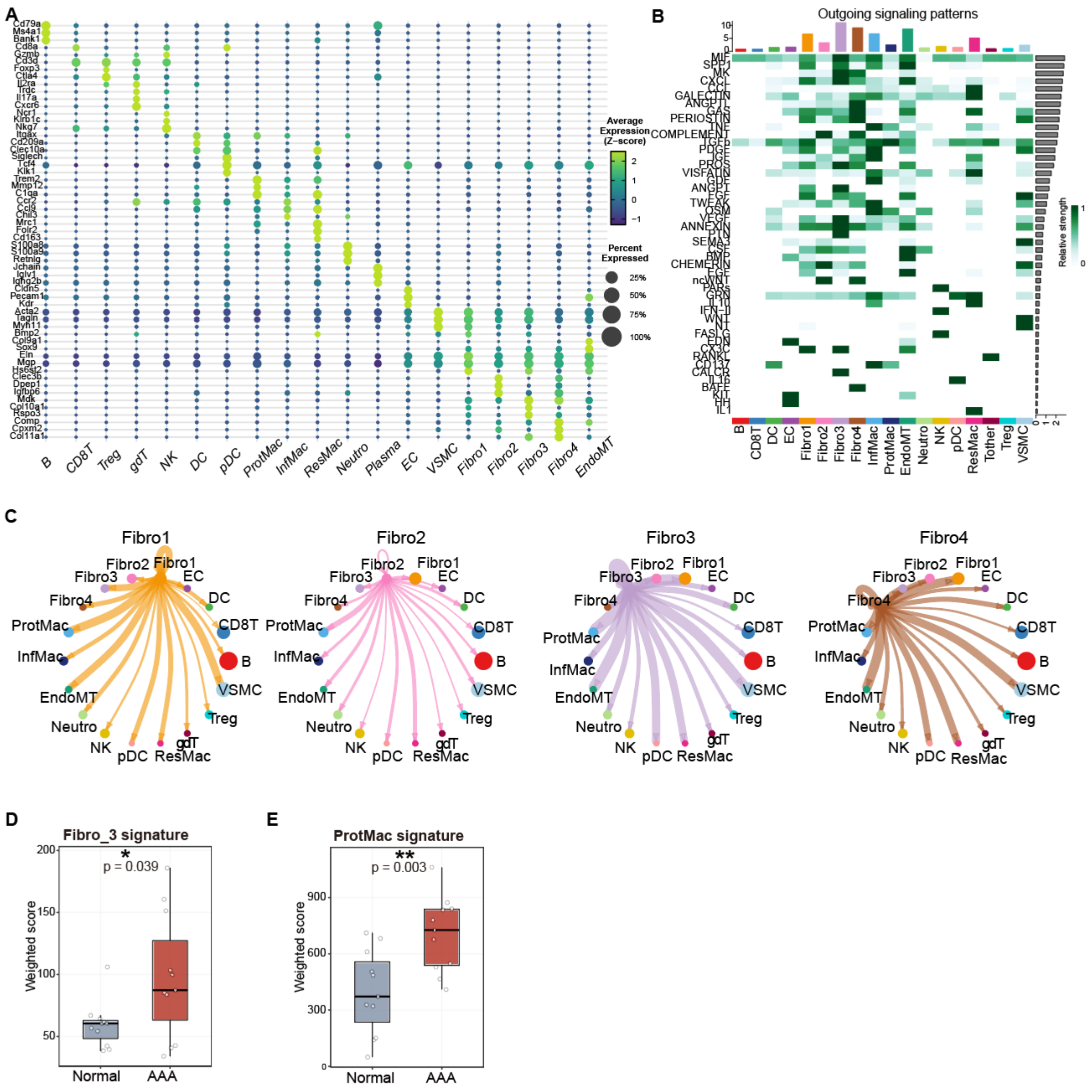

Supplementary Fig. S4. Extended characterization of the COL10A1⁺ fibroblast communication network (Related to Figure 5).

(A) Dot plot displaying the top three marker genes for each major cell population annotated in Figure 5A.

(B) Heatmaps showing the relative signaling strength across all cell populations within major signaling pathways. Color gradient from blue to white indicates decreasing interaction strength.

(C) Circle plots illustrating the outgoing communication intensity from each fibroblast subpopulation to all other cell types. Line thickness represents interaction strength.

(D–E) Weighted signature scores of Fibro_3 and ProtMac in AAA (n = 11) versus normal controls (n = 11). High-specificity marker genes were identified from mouse

scRNA-seq data (pct.1 > 0.5, pct.2 < 0.1, avg_log2FC > 1.0), mapped to human orthologs, and used to compute weighted signature scores based on original logFC values in human bulk RNA-seq data. Two independent datasets (GSE202267 and GSE183464) were integrated using ComBat batch correction. Statistical significance was assessed using Welch's two-tailed t-test. Data are presented as mean ± SD. *, $p < 0.05$; **, $p < 0.01$.
